# Biochemical characterization of INTS3 and C9ORF80, two subunits of hNABP1/2 heterotrimeric complex in nucleic acid binding

**DOI:** 10.1042/BCJ20170351

**Published:** 2018-01-02

**Authors:** Venkatasubramanian Vidhyasagar, Yujiong He, Manhong Guo, Tanu Talwar, Ravi Shankar Singh, Manisha Yadav, George Katselis, Franco J. Vizeacoumar, Kiven E. Lukong, Yuliang Wu

**Affiliations:** 1 Department of Biochemistry, University of Saskatchewan, Health Sciences Building, 107 Wiggins Road, Saskatoon, Saskatchewan, Canada S7N 5E5; 2 Department of Medicine, University of Saskatchewan, Health Sciences Building, 107 Wiggins Road, Saskatoon, Saskatchewan, Canada S7N 5E5; 3 Canadian Centre for Health and Safety in Agriculture, University of Saskatchewan, Health Sciences Building, 107 Wiggins Road, Saskatoon, Saskatchewan, Canada S7N 5E5; 4 Saskatchewan Cancer Agency, University of Saskatchewan, Health Sciences Building, 107 Wiggins Road, Saskatoon, Saskatchewan, Canada S7N 5E5

**Keywords:** C9ORF80, DNA binding, INTS3, NABP1, NABP2, RNA-binding proteins

## Abstract

Human nucleic acid-binding protein 1 and 2 (hNABP1 and hNABP2, also known as hSSB2 and hSSB1 respectively) form two separate and independent complexes with two identical proteins, integrator complex subunit 3 (INTS3) and C9ORF80. We and other groups have demonstrated that hNABP1 and 2 are single-stranded (ss) DNA- and RNA-binding proteins, and function in DNA repair; however, the function of INTS3 and C9OFR80 remains elusive. In the present study, we purified recombinant proteins INTS3 and C9ORF80 to near homogeneity. Both proteins exist as a monomer in solution; however, C9ORF80 exhibits anomalous behavior on SDS–PAGE and gel filtration because of 48% random coil present in the protein. Using electrophoretic mobility shift assay (EMSA), INTS3 displays higher affinity toward ssRNA than ssDNA, and C9ORF80 binds ssDNA but not ssRNA. Neither of them binds dsDNA, dsRNA, or RNA : DNA hybrid. INTS3 requires minimum of 30 nucleotides, whereas C9OFR80 requires 20 nucleotides for its binding, which increased with the increasing length of ssDNA. Interestingly, our GST pulldown results suggest that the N-terminus of INTS3 is involved in protein–protein interaction, while EMSA implies that the C-terminus is required for nucleic acid binding. Furthermore, we purified the INTS3–hNABP1/2–C9ORF80 heterotrimeric complex. It exhibits weaker binding compared with the individual hNABP1/2; interestingly, the hNABP1 complex prefers ssDNA, whereas hNABP2 complex prefers ssRNA. Using reconstituted heterotrimeric complex from individual proteins, EMSA demonstrates that INTS3, but not C9ORF80, affects the nucleic acid-binding ability of hNABP1 and hNABP2, indicating that INTS3 might regulate hNABP1/2's biological function, while the role of C9ORF80 remains unknown.

## Introduction

Although DNA primarily exists as a duplex, there are many cellular processes like replication and transcription, where single-stranded DNA (ssDNA) is exposed, making it more vulnerable to damage and enzymatic degradation. Single-stranded DNA-binding proteins (SSBs) protect these structures from nuclease attack, and also recruit appropriate proteins like DNA repair enzymes and cell cycle regulatory proteins [1,2]. SSB proteins have a characteristic domain, oligonucleotide/oligosaccharide binding (OB)-fold, which facilitates binding to nucleic acid, as well as protein–protein interactions [3]. Replication protein A (RPA), a major SSB in human that functions as heterotrimers (RPA70, 32, and 14), has multiple OB-folds and essentially functions at various cellular processes such as DNA replication, repair, and recombination [4,5]. Human nucleic acid-binding protein 1 and 2 (hNABP1 and hNABP2, also known as hSSB2 and hSSB1) are two newly identified SSB [6] that has one OB-fold.

Many studies have shown that both hNABP1 and hNABP2 form an independent complex with two proteins, INTS3 (integrator complex subunit 3) and C9ORF80 [7–9]. INTS3 is a member of the Integrator complex that is characterized as an RNA polymerase II (RNAPII) C-terminal domain-binding factor, and is involved in the 3′ processing of small nuclear RNAs (snRNAs) [10]. The Integrator complex contains at least 14 different subunits (named INTS1–14) whose individual contributions to snRNA processing or broader cellular functions that remain largely uncharacterized [11]. By size-exclusion chromatography, the entire mass of the complex is greater than 1 MDa, since most of INTS's molecular mass is >100 kDa. The most common motifs within INTSs are α-helical repeats (HEAT, ARM, and TPR or VWA domains), suggestive of protein–protein interaction surfaces. Among the complex, INTS9 and 11 are homologous of CPSF100 (Cleavage and polyadenylation specificity factor 100 kDa subunit) and CPSF73, which are essential for 3′-end cleavage of RNAPII-transcribed messenger RNAs [12]. INTS1 and INTS5 are required for snRNA processing [13,14], INTS6 forms a stable complex with INTS3 and participates in DNA damage response [9], but the function of other INTS proteins remains largely unexplored. On the other hand, C9ORF80 is a 104-residue polypeptide and has never been characterized.

The structure of INTS3–hNABP2–C9ORF80 reveals that INTS3 acts as a scaffold to bridge hNABP2 and C9ORF80; however, only the N-terminus of INTS3 (1–500 amino acids) was used in this study [15]. It has been shown that INTS3 is essential for the expression as well as recruitment of hNABP2 at double-stranded DNA breaks (DSBs), and also required for efficient homologous recombination (HR)-dependent repair [16,17]. While the depletion of INTS3 has significantly affected the expression and IR-induced foci formation of hNABP2 [7,8,16]. Zhang et al. [17] have shown that the N-terminus of INTS3 is involved in the interaction with the OB-fold of hNABP2. Nearly 300 amino acid deletions from the C-terminus or N-terminus of INTS3 have significantly decreased the IR-induced foci formation of INTS3, and deletion of both ends abolished the foci formation completely, suggesting that both N-terminus and C-terminus of INTS3 are essential. Loss of C9ORF80, on the other hand, does not affect the expression of hNABP2, rather only partially affects the expression of INTS3 [8].

Despite hNABP1/2, especially hNABP2, has been studied extensively, the biochemical function of INTS3 and C9ORF80 remains to be elucidated. In the present study, we purified the recombinant proteins INTS3 and C9ORF80 to near homogeneity. Although both proteins exist as monomers, C9ORF80, but not INTS3, exhibits anomalous behavior in gel filtration and SDS–PAGE because of random coil conformation. INTS3 has higher affinity toward ssRNA than ssDNA, and C9ORF80 exhibited ssDNA binding but not with ssRNA. The C-terminus of INTS3 is required for its nucleic acid binding, while the N-terminus is involved in protein–protein interactions. In addition, we have also successfully purified the INTS3–hNABP1/2–C9ORF80 complex and found it also exhibited similar substrate affinity like hNABP1/2 alone; however, the complex has reduced affinity, which is because of INTS3 but not C9ORF80.

## Experimental

### Plasmids

Human cDNA clones of INTS3 and C9ORF80 were purchased from the SPARC BioCentre, The Hospital for Sick Children, Toronto, Canada. The *INTS3* (full length, N-terminus, and C-terminus) were amplified and cloned into the *BamH*I and *Xho*I sites of pGEX-6P-1 vector (GE Healthcare). The C-terminus of *INTS3* was amplified and cloned into the *Nde*1 and *Xho*1 sites of pET28a vector (Novagen). The C9ORF80 was amplified and cloned into the *Nco*I and *Xho*I sites of pET28a vector. All plasmids were verified by DNA sequencing. The primers used for the amplification of genes are listed in Supplementary Table S1. The vector construction of hNABP1/2 were described recently [18].

### Proteins

For INTS3 full-length (INTS3^FL^) expression and purification, the *Escherichia coli* Rosetta 2 cells were transformed with pGEX-6P-1-INTS3 plasmid and the transformants were grown in LB medium containing 0.3% glucose, 100 μg/ml of ampicillin, and 34 μg/ml of chloramphenicol at 37°C. After *A*_600_ reaching 0.6, the protein expression was induced by adding IPTG to the final concentration of 0.5 mM and incubated at 15°C for 16 h, and the cells were harvested by centrifugation at 5000 ***g*** for 10 min at 4°C and stored at –80°C until use. The cells were suspended in buffer A (25 mM Tris–HCl, pH 8.0, 0.5 M NaCl, 1 mM DTT, 1 mM EDTA, 0.2% Tween 20 and 10% glycerol) and 1 mM phenylmethylsulfonyl fluoride (PMSF), lysed by sonication (5 cycles of 10 s pulse with 1 min interval), and the soluble fraction was obtained by centrifugation at 25 000 ***g*** for 30 min at 4°C. The supernatant having GST-INTS3 was incubated with 2 ml of glutathione-agarose beads (GE Healthcare) for 3 h at 4°C, and the beads were washed with 10 column volume (CV) of buffer B (25 mM Tris–HCl, pH 8.0, 0.15 M NaCl, 1 mM DTT, 0.2% Tween 20, and 10% glycerol) containing 2 mM MgSO_4_. The GST-tag was cleaved by incubating the beads with 1 ml of buffer B consisting of 1 mM EDTA and PreScission Protease (10 units/ml, NEB) for overnight at 4°C. The fractions were eluted with three CVs of buffer A, electrophoresed on 8% SDS–PAGE gel, and stained with Coomassie Blue to determine their purity. The pure fractions were pooled and loaded onto a Sephacryl S-300 HR column pre-equilibrated with buffer B consisting of 50 mM of l-arginine and 50 mM of glutamic acid (Sigma), and the proteins were eluted at the flow rate of 0.5 ml/min with the same buffer used for calibration. The protein was confirmed with SDS–PAGE, and the fractions were pooled and concentrated. The N- and C-terminal GST-tagged INTS3s (INTS3^N^ and INTS3^C^, respectively) were also purified similarly.

Expression and purification of C9ORF80 protein was done based on Richard et al. procedure [6] with some modifications. Briefly, recombinant C9ORF80 proteins were expressed in *E. coli* Rosetta 2 cells and subjected to a two-step purification using Nickel Affinity Gel (Sigma) and Sephacryl S-100 HR chromatography. The Rosetta 2 cells harboring the recombinant gene were grown at 37°C in LB medium containing 50 μg/ml kanamycin and 34 μg/ml of chloramphenicol until the *A*_600_ reached 0.6. The protein expression was induced by adding IPTG to the final concentration of 0.5 mM with extended incubation for 16 h at 15°C, and the cells were harvested by centrifugation at 5000 ***g*** for 10 min at 4°C and stored at −80°C until used. The cells were lysed by sonication in buffer C (25 mM Tris, pH 8.0, 0.15 M NaCl, 100 µM Tween 20, and 10% glycerol) with 1 mM PMSF. The cell debris and inclusion bodies were removed by centrifugation at 45 000 ***g*** for 30 min at 4°C. The supernatant was applied to Ni-NTA beads equilibrated with buffer C, washed with 10 CV of buffer D (25 mM Tris, pH 8.0, 0.5 M NaCl, 100 µM Tween 20, and 10% glycerol) containing 25 mM imidazole, and eluted with 5 CV of buffer D containing 250 mM imidazole. The protein fractions were confirmed with SDS–PAGE; fractions with high protein yield were pooled and subjected to size-exclusion chromatography on a Sephacryl S-100 HR 16/60 column (GE Healthcare) equilibrated with buffer C containing 0.1% β-mercaptoethanol. The fractions were collected at a flow rate of 0.5 ml/min in buffer C. The protein was confirmed with SDS–PAGE, and the fractions were pooled and concentrated. The His-tagged INTS3^C^ protein was purified similarly, except the gel filtration buffer B has 50 mM of l-arginine and 50 mM of glutamic acid (Sigma). The hNABP1 and hNABP2 proteins were purified as described recently [18].

For the heterotrimeric complex expression and purification, the *INTS3* gene was cloned in a pGEX-6P-1 vector, the *hNABPs* in pET29a, and the *C9ORF80* in pET28a, and were co-transformed into *E. coli* Rosetta 2 cells. Bacteria harboring the plasmids were grown in LB medium containing 50 μg/ml of ampicillin, 15 μg/ml of kanamycin, and 34 μg/ml of chloramphenicol until the *A*_600_ reached 0.6; the protein expression was induced by the addition of IPTG to the final concentration of 0.1 mM. After incubation for 16 h at 15°C, the cells were harvested by centrifugation at 5000 ***g*** for 10 min and lysed by sonication in buffer A. The INTS3–hNABP–C9ORF80 complex in the soluble fraction was purified using glutathione-agarose beads, followed by Ni-NTA beads as described earlier. The fractions were electrophoresed on 8% SDS–PAGE using Tricine buffer [19].

### Sucrose density gradient centrifugation

A linear sucrose gradient (8–20%, wt/vol) was prepared in a buffer B using 11× 60-mm centrifugation tubes (Beckman). The gradients were stored at least 1 h at 4°C before they were loaded with INTS3^FL^ protein fractions eluted from gel filtration chromatography (0.5 mg/ml; 100 µl) and centrifuged at 120 000 ***g*** for 16 h in an SW Ti60 rotor (Beckman) at 4°C. After centrifugation, fractions of 100 µl were collected from the top and analyzed by 8% SDS–PAGE. Standard globular proteins (carbonic anhydrase, 29 kDa; bovine serum albumin, 66 kDa; and alcohol dehydrogenase, 150 kDa) were run in parallel. The molecular mass of INTS3^FL^ protein was calculated using the formula *M* = *fS*Na/(1 − *νρ*), where *f* is the Stokes radius of protein, *S* is the Svedberg unit of the protein, Na is the Avogadro's number, *ν* is the specific volume of the protein, and *ρ* is the density of solution.

### Size-exclusion chromatography coupled with multi-angle light-scattering

Size-exclusion chromatography with multi-angle light-scattering (SEC-MALS) was performed using the Waters HPLC systems coupled with the refractive index and static laser light-scattering instruments (Wyatt MiniDAWN Treos and Wyatt OptiLab rEX, respectively). The gel filtration fractions corresponding to C9ORF80 protein were concentrated using 3 kDa membrane cut-off and injected (500 µl, 4.2 mg/ml) into the SEC 70 column (Bio-Rad, 10 × 300 mm, 24 ml, 500–70 000 kDa) equilibrated with buffer D. The light-scattering and refractive index data were used to calculate the weight-averaged molar mass and the mass fraction of each peak using the Astra™ package v. 6.1.2 (Wyatt Technologies).

### Antibody

Using the previously purified full-length hNABP1 and hNABP2 proteins [18], rabbit polyclonal antisera were made by the GenScript. The goat polyclonal antibody against INTS3 (sc-138355) and rabbit polyclonal antibody against C9ORF80 (sc-137357) were purchased from Santa Cruz.

### Western blotting

The purified INTS3 protein (1 μg) was separated on 8% SDS–polyacrylamide gel using Laemmli buffer, while C9OFR80 was separated on 8 or 10% SDS–polyacrylamide gel using Tricine buffer. After electrophoresis, the proteins were transferred onto the nitrocellulose membrane (Bio-Rad) at 150 V for 2 h at 4°C. The membrane was incubated with blocking buffer (5% skimmed milk in PBST buffer) for 1 h at room temperature. Then, the membrane was incubated with goat polyclonal antibody against INTS3 (sc-138355, Santa Cruz) or rabbit polyclonal antibody against C9ORF80 (sc-137357, Santa Cruz), at 1 : 1000 dilution in PBST, consisting of 1% skimmed milk, at 4°C for overnight. After washing with PBST for five times (5 min each), the membrane was incubated with donkey anti-goat (for INTS3) or goat anti-rabbit (C9ORF80) IgG-HRP antibody (1 : 10 000 in PBST consisting of 1% skimmed milk, Santa Cruz) for 1 h at room temperature and washed with PBST for five times (5 min each). The membrane was then treated with ECL reagent (GE Healthcare) according to the manufacturer's instruction.

### Circular dichroism spectroscopy

The INTS3 and C9ORF80 proteins purified as mentioned above were applied to Sephacryl S-300 and Sephacryl S-100 HR 16/60 columns, respectively, and eluted with buffer E (10 mM phosphate buffer, pH 7.4, and 100 mM NaCl), and concentrated. The CD (circular dichroism) spectra for these proteins (1 mg/ml) were acquired using Chirascan plus (Applied Photophysics) in 1 mm cuvettes, and the spectrum was recorded between 190 and 260 nm. Baselines were adjusted with buffer E, scanned 10 times, and averaged. The proteins were also scanned 10 times and averaged. The averaged baseline was subtracted from the averaged sample spectrum. The secondary structure content was analyzed using DichroWeb [20] with respective spectrum input.

### GST pulldown

The bacterial-expressed and -purified GST-INTS3^FL^, -INTS3^N^, or -INTS3^C^ were incubated with hNABP1/2 or C9ORF80 independently. The mixture was incubated at 4°C for 1 h with gentle shaking, followed by another hour of incubation with 100 µl glutathione-agarose beads (Sigma). The beads were washed several times with lysis buffer to remove excess proteins, and the proteins bound were eluted using 10 mM glutathione (Sigma). The proteins were resolved on 8% SDS–PAGE and detected by Western blotting.

### Oligonucleotides and substrates

PAGE-purified oligonucleotides were purchased from Integrated DNA Technologies and are listed in Supplementary Table S1. Oligonucleotide was 5′-end-labeled with [γ-^32^P] ATP using T4 polynucleotide kinase (NEB) at 37°C for 1 h. Unincorporated radionucleotides were removed by a G25 chromatography column (GE Healthcare). The labeled ssDNA or ssRNA substrates were kept at 4°C and ready to use. For the dsDNA substrate, a [γ-^32^P] ATP-labeled oligonucleotide was annealed to a 2.5-fold excess of the unlabeled complementary strands in annealing buffer (10 mM Tris–HCl, pH 7.5, and 50 mM NaCl) by heating at 95°C for 6 min and then cooling slowly to room temperature. For dsRNA and RNA : DNA hybrid, [γ-^32^P] ATP-labeled oligonucleotide was annealed to a 2.5-fold excess of the unlabeled complementary strands in annealing buffer (10 mM MOPS, pH 6.5, 1 mM EDTA, and 50 mM KCl) by heating at 95°C for 6 min and then cooling slowly to room temperature. All double-stranded substrates were purified by PAGE isolation, and their concentrations were determined by liquid scintillation counting before use.

### Electrophoretic mobility shift assays

Protein/nucleic acid-binding mixtures (20 μl) contained the indicated concentrations of protein (hNABP1, hNABP2, INTS3, and C9ORF80) and 0.5 nM of the specified ^32^P-end-labeled DNA or RNA substrate in buffer (20 mM HEPES, pH 7.3, 100 mM KCl, 1 mM MgCl_2_, 1 mg/ml BSA (bovine serum albumin), 1 mM DTT, and 6% glycerol). The binding mixtures were incubated on ice for 30 min after the addition of proteins. After incubation, 3 μl of loading dye (74% glycerol, 0.01% xylene cyanol, and 0.01% bromophenol blue) was added to each mixture, and the samples were resolved in native 5% polyacrylamide gels (19 : 1 acrylamide : bisacrylamide) using 1× TBE buffer at 200 V for 2 h at 4°C. The radiolabeled species were visualized using a Phosphor-Imager and analyzed with ImageQuant software (GE Healthcare). The dissociation constant (*K*_d_) was determined using the following equation *K*_d_ = [A][B]/[AB], where [A], [B], and [AB] are the concentrations of proteins, nucleic acid, and protein–nucleic acid complex, respectively. However, as the concentration of nucleic acid (B) is very low when compared with that of proteins, the concentration of protein [A] used to shift 50% of nucleic acid ([AB] = [B], thus [B]/[AB] = 1) was used to determine the *K*_d_ [21]. The shift is expressed as percentage (%) which is the ratio of shifted band and substrate band.

### Immunoprecipitation and mass spectrometry

HeLa cells collected from 2 × 150 cm dish were resuspended in 5 ml of immunoprecipitation (IP) buffer (20 mM Tris, pH 7.5, 150 mM KCl, 5% glycerol, 0.5 mM EDTA, and 0.5% NP-40) supplemented with protease inhibitor cocktail and 2 µM PMSF. The lysate was centrifuged at 100 000×***g*** for 30 min at 4°C, and supernatant was collected. The supernatant was divided into equal two parts, and hNABP2 antibody or rabbit IgG was added and incubated for 1 h at 4°C. Following this, protein A beads were added, incubated for overnight at 4°C, and beads were collected by centrifugation at 500×***g*** for 5 min. The beads were washed five times with IP buffer and proteins were eluted by incubation in 2× SDS loading dye at 80°C for 5 min. The eluted samples were separated on 10% SDS–PAGE gel using the Tricine buffer system. After visualized with silver staining (cat#24600, Thermo Fisher), the bands that were present in hNABP2 pulldown, but absent from control IgG, were excised from the gel and destained as per the manufacturer's instruction, followed by in-gel digestion with trypsin. The samples were injected onto an Agilent Zorbax 300SB C8 (40 nl enrichment column; 75 μm × 43 mm separation column; 5 μm; Agilent Technologies) custom-made chip for desalting and online mass spectrometric analysis. The extracted samples were loaded onto the enrichment column using 95% mobile phase A (97% water, 0.1% formic acid, and 3% acetonitrile) and 5% mobile phase B (10% water, 0.1% formic acid, and 90% acetonitrile) at a flow rate of 4 μl/min. Positive ESI mass spectra were acquired in 3200 *m/z*, 2 GHz extended dynamic range mode. The data extraction and protein confirmation were done using the Agilent MassHunter BioConfirm software and searched against the Swiss-Prot Human database. The search parameters included trypsin digestion, carbamidomethyl of cysteine residues as fixed modification, and methionine oxidation as variable modification.

## Results

### Full-length INTS3 protein exists as a monomer in solution

**Figure 1.**
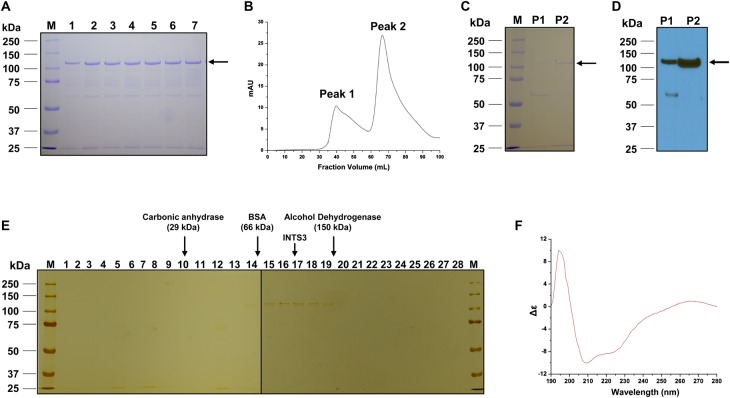
Purification of full-length INTS3 protein. (**A**) SDS–PAGE analysis of INTS3^FL^ protein eluted from glutathione resin digested with PreScission protease. M, marker; 1–7, fractions collected. (**B**) Chromatographic profile of the recombinant INTS3^FL^ protein eluted from a Sephacryl S-300 HR column. Peaks are indicated. (**C**) SDS–PAGE analysis of the gel filtration-eluted INTS3^FL^ fractions. P1, peak 1; P2, peak 2. (**D**) Western blot analysis of the purified proteins (shown in **C**) using an anti-INTS3 antibody. (**E**) Silver staining of INTS3^FL^ fractions from the sucrose gradient centrifugation. Thirty microliter for each fractions (1–28) was loaded per lane. The positions of standards are indicated at the top. (**F**) CD spectrum of INTS3^FL^ protein.

The N-terminal fragment of INTS3 (1–500 aa) has been purified from *E. coli* [15], and its full-length has been purified from insect cells with poor yield [7]. We cloned *INTS3^FL^* into a pGEX-6P1 vector, and the expressed protein was purified by affinity and size-exclusion chromatography. The fractions obtained from glutathione sepharose beads (Figure 1A) were pooled and loaded on a Sephacryl S-300 HR column; however, a large portion of proteins were aggregated and eluted in the void volume of the column, while a minor fraction eluted corresponding to monomer (data not shown). To reduce the aggregation, we added arginine and glutamic acid (50 mM each) into the elution buffer [22,23] and observed a minor peak (peak 1) and a major peak (peak 2) corresponding to the elution volume (Ve) of ∼40 ± 2 and ∼69 ± 2.5 ml, respectively (Figure 1B). Electrophoresis of the fractions from both peaks on SDS–polyacrylamide gel showed similar migration bands (Figure 1C), with two bands corresponding to ∼120 and 60 kDa; however, the protein corresponding to ∼60 kDa was intense in peak 1. Based on the molecular mass calibration of the Sephacryl S-300 HR column (Supplementary Figure S1A), peak 1 from the void volume probably represents aggregation of INTS3^FL^ or its degraded protein, while peak 2 corresponds to the molecular mass of 113 ± 18 kDa, which is close to the predicted molecular mass of INTS3^FL^ (118.01 kDa). The identity of the INTS3 protein was further confirmed by Western blot using an anti-INTS3 antibody (Figure 1D) and mass spectrometry analysis (Supplementary Table S2). To further confirm the molecular mass of INTS3^FL^ protein in peak 2, we performed sucrose density gradient centrifugation. SDS–PAGE analysis showed that INTS3^FL^ protein migrated between bovine serum albumin (66 kDa) and alcohol dehydrogenase (150 kDa) (Figure 1E and Supplementary Figure S1B). The molecular mass of INTS3^FL^ was calculated as ∼122 ± 4 kDa. To determine whether INTS3^FL^ protein has folded correctly, a CD spectrum was performed. The far UV-wavelength scan of INTS3^FL^ protein exhibited that the protein was well folded with predominant α-helical structures, having a dip at 222 and 208 nm (Figure 1F). Analysis of the CD data with Selcon 3 [24] has revealed that the INTS3^FL^ has 58% α-helices, 5% β-sheets, and 37% disordered. The secondary structure predicted by PSIPRED (PSI-blast-based secondary structure PREDiction) [25] has also shown similar results: 55% α-helices, 2% β-sheets, and 43% random coil (Supplementary Figure S2). Also, we consistently noted an additional band at ∼60 kDa on SDS–PAGE (Figure 1A,C). Mass spectrometric analysis of this band revealed it was the N-terminal fragment of INTS3 (Supplementary Table S3). Thus, we conclude that the INTS3^FL^ protein in peak 2 exists as a monomer in solution, and we used this fraction for our further experiments.

### Full-length INTS3 protein binds ssDNA and ssRNA

**Figure 2.**
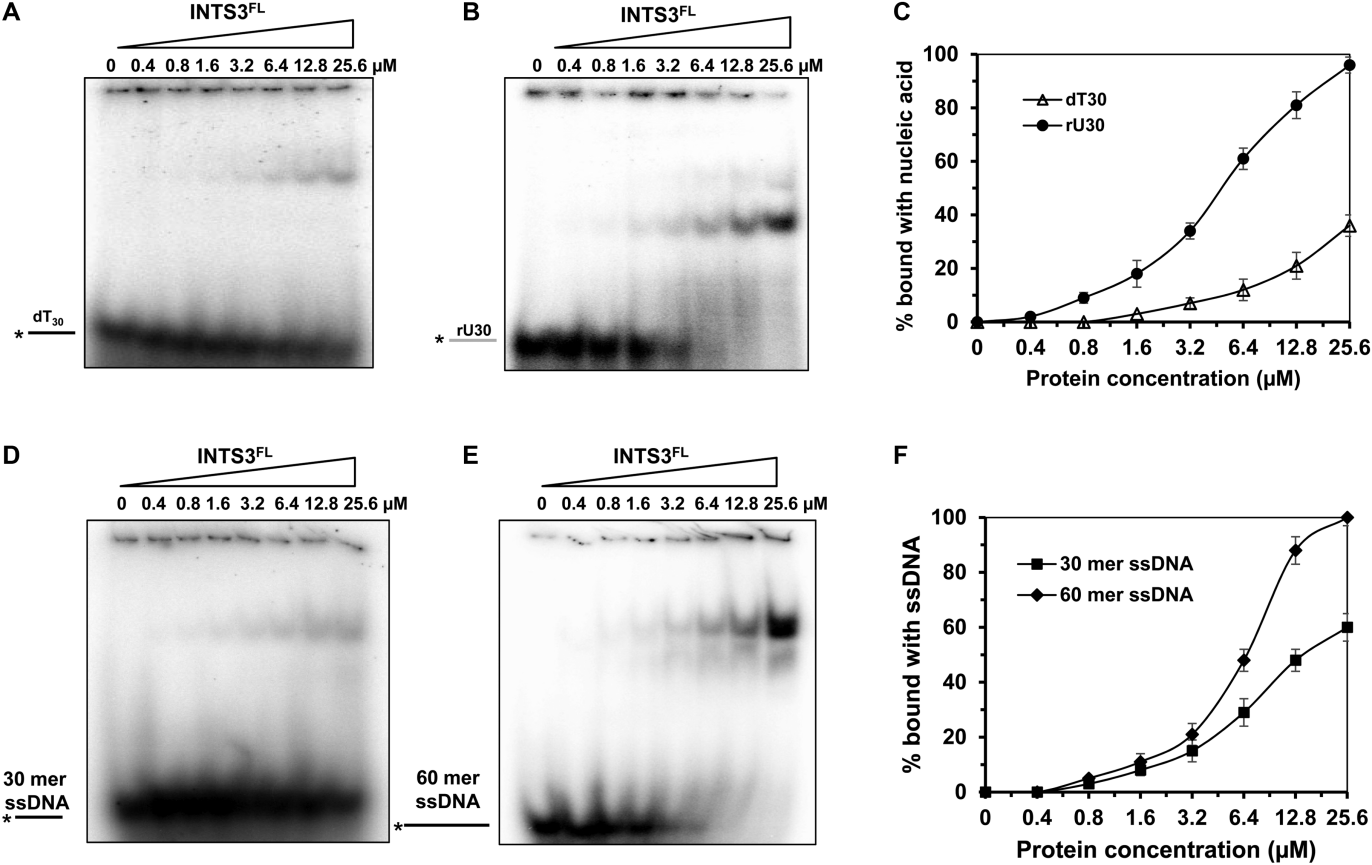
Nucleic acid binding of full-length INTS3 protein. Representative EMSA images for INTS3^FL^ protein incubated with 0.5 nM of ssDNA dT_30_ (**A**) and ssRNA rU_30_ (**B**). DNA is in black, and RNA in gray. (**C**) Quantitative analyses for INTS3^FL^ with nucleic acids shown in (**A** and **B**). Data represent the mean of three independent experiments with standard deviation (SD) indicated by error bars. Representative EMSA images for INTS3^FL^ protein incubated with 0.5 nM of random 30-mer ssDNA (**D**) and random 60-mer ssDNA (**E**). (**F**) Quantitative analyses for INTS3^FL^ with nucleic acids shown in (**D** and **E**).

The nucleic acid-binding ability of NABP1/2 has been well documented [6,18]; however, the characteristics of INTS3^FL^ have never been reported. Thus, to understand the nucleic acid-binding ability of INTS3^FL^, we incubated the protein with ssDNA (dT_30_) and ssRNA (rU_30_). We found that INTS3^FL^ could bind both dT_30_ (Figure 2A) and rU_30_ (Figure 2B); however, INTS3^FL^ had higher affinity toward rU_30._ The protein could bind dT_30_ at the concentration of 1.6 µM and exhibited 36% binding at 25.6 µM, though it is a physiologically irrelevant concentration (Figure 2C). However, it could bind rU_30_ at the concentration of 0.4 µM and displayed 96% of binding at the concentration of 25.6 μM with *K*_d_ value of 5.6 ± 1.2 µM (Figure 2C). It is not unusual for INTS3 to exhibit higher binding toward rU30, since the integrator complex is involved in RNA processing [10]. Since INTS3 showed higher substrate binding toward rU_30_ than dT_30_, we then evaluated the binding of random 30-mer ssRNA. However, the protein exhibited weak binding with random ssRNA, 5% binding at 12.8 µM (Supplementary Figure S3A), while it exhibited 81% binding with rU_30_ at 12.8 µM (Figure 2C), suggesting a robust sequence specificity toward ssRNA.

As hNABP1/2 binds effectively with increasing length of ssDNA [18], next we assessed the binding ability of INTS3^FL^ with various lengths of ssDNA such as dT_10_, dT_20_, dT_60_, and dT_90_. We found that INTS3^FL^ requires a minimum of 30 nucleotides and its affinity increases with the length of ssDNA (Supplementary Figure S3B). We observed two and three shifted bands with 60- and 90-mer oligo (dT), suggesting that INTS3 can bind 30 nucleotides in an orderly manner. We also examined INTS3^FL^ binding with a random ssDNA sequence and found that the protein bound these nucleotides. Similarly, the affinity increased with the length of ssDNA (30-mer and 60-mer, Figure 2D,E). At the concentration of 25.6 µM, the protein exhibited 58% binding with 30-mer random ssDNA with a *K*_d_ value of 13 ± 0.8 µM, and 100% binding with 60-mer random ssDNA with a *K*_d_ value of 6.4 ± 0.9 µM (Figure 2F). However, INTS3 did not bind dsRNA (Supplementary Figure S3C), dsDNA (Supplementary Figure S3D), or DNA : RNA hybrid (Supplementary Figure S3E).

### C9ORF80 protein is monomeric in solution and disordered

**Figure 3.**
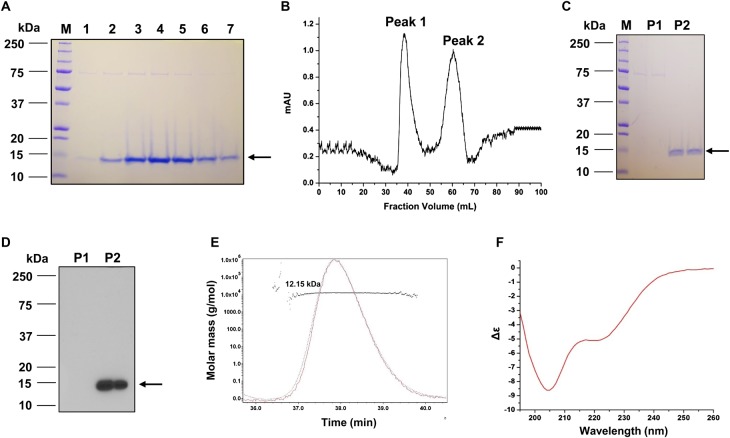
Purification of C9ORF80 protein. (**A**) SDS–PAGE analysis of C9ORF80 protein eluted from Ni-NTA beads. 1–7, fractions collected. (**B**) Chromatographic profile of the recombinant C9ORF80 protein eluted from a Sephacryl S-100 HR column. Peaks are indicated. (**C**) SDS–PAGE analysis of the gel filtration-eluted C9ORF80 fractions. Two lanes for each peak. P1, peak 1; P2, peak 2. (**D**) Western blot analysis for the purified proteins (shown in **C**) using an anti-C9ORF80 antibody. (**E**) SEC-MALS analysis of C9ORF80 protein shows the average molecular mass of the protein peak and the variation across the peak. (**F**) CD spectrum of C9OFR80 protein.

The property of C9ORF80 has never been characterized. To understand the function of C9ORF80, we cloned *C9ORF80* gene into a pET28a vector and overexpressed in bacteria. The fractions from affinity chromatography had the target C9ORF80 protein (near 15 kDa) along with an unknown protein of molecular mass of 70 kDa (Figure 3A). We pooled the fractions and applied them to a Sephacryl S-100 HR column, and observed that there were two peaks: peak 1 (∼39 ml) and peak 2 (∼62 ml) (Figure 3B). Electrophoresis of the gel filtration chromatography fractions from two peaks on SDS–polyacrylamide gel showed two migration bands corresponding to the unknown protein of 70 kDa (peak 1) and C9ORF80 (peak 2) protein corresponding to the molecular mass of ∼15 kDa (Figure 3C). According to the molecular mass standards used to calibrate the size-exclusion column (Supplementary Figure S1C), the molecular mass of C9ORF80, peak 2, was similar to the globular protein molecular mass of 22 ± 4 kDa, while the peak 1 was in the void volume. This peak 1 corresponding to 70 kDa protein has also been observed in other bacterial purifications, such as yeast Mtw1 and human DDX41 [26,27], and they suggest that it might be DnaK (Hsp70), a chaperone protein that assists in protein folding. The identity of the C9ORF80 protein was further confirmed by Western blot using an anti-C9ORF80 antibody (Figure 3D) and mass spectrometry analysis (Supplementary Table S4). To determine the molecular mass of C9ORF80 protein directly, we performed MALS and refractive index measurements of the fractions collected from peak 2. The results revealed that the molecular mass of the protein is 12.15 ± 1.2 kDa (with His-tag; Figure 3E), which is close to the predicted size, 12.4 kDa (with His-tag). Since the additional 70 kDa protein was not observed in peak 2 of the gel filtration fraction and not detected by the anti-C9ORF80 antibody, we used this fraction for our further experiments.

Although the predicted molecular mass of C9ORF80 is 11.44 kDa, the molecular mass in size-exclusion chromatography (22 kDa) and SDS–PAGE (∼15 kDa) is much higher than predicted. Thus, we evaluated the secondary structure of C9ORF80 with PSIPRED [25] and PONDR (predictor of natural disordered regions) [28]. The predication by PSIPRED (Supplementary Figure S4A) and PONDR (Supplementary Figure S4B) suggests that ∼45% of C9ORF80 protein exists as random coil. To confirm this, we determined the secondary structure of protein using CD spectroscopy (Figure 3F), where the protein exhibited a minimal ellipticity, negative bands near 200 nm corresponding to random coil conformation, and a dip at 208 nm suggesting α-helix. When analyzed with K2d method [29] using the DichroWeb software [20], 48% of the protein exhibits random coil, 37% corresponds for α-helix, and 15% with β-sheets, which is close to the results predicted. Therefore, we conclude that C9ORF80 exists as a monomer in solution, but has more random coils, and exhibits anomalous behavior in gel filtration and SDS–PAGE.

### C9ORF80 binds ssDNA but not ssRNA

**Figure 4.**
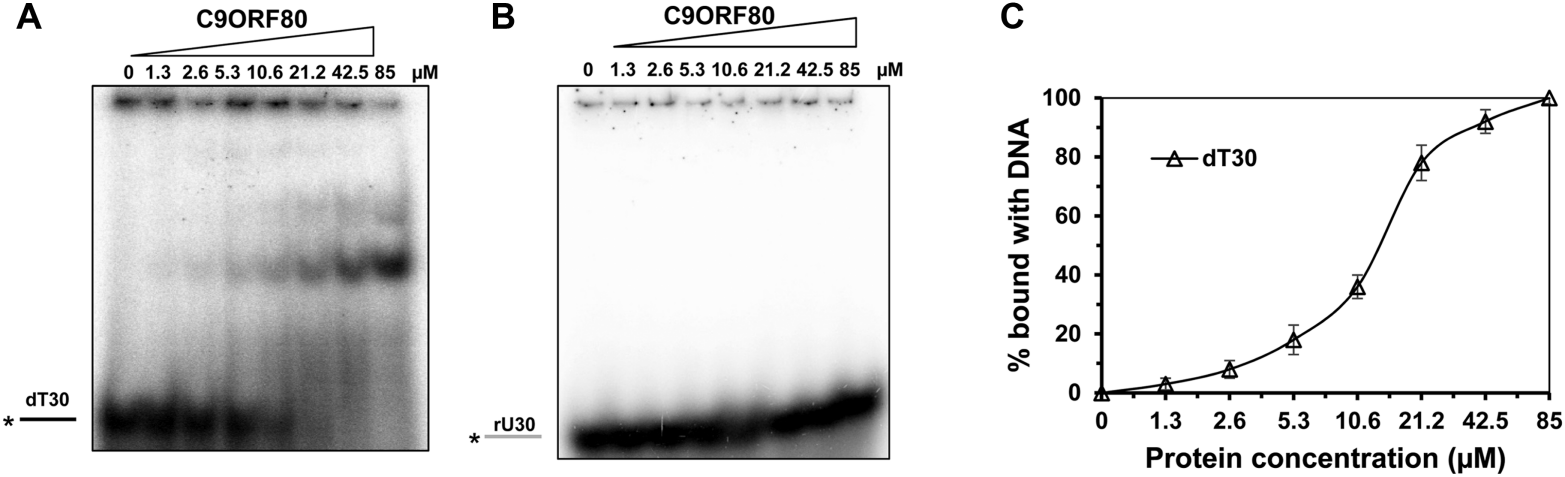
Nucleic acid binding of C9ORF80 protein. Representative EMSA images for C9ORF80 protein incubated with 0.5 nM ssDNA dT_30_ (**A**) and ssRNA rU_30_ (**B**). (**C**) Quantitative analyses for C9ORF80 binding with nucleic acid shown in (**A**).

Similar to INTS3, the nucleic acid-binding activity of C9ORF80 protein has not been studied. Using dT_30_ and rU_30_, we found that C9ORF80 could bind with dT_30_ but barely with rU_30_ substrate (Figure 4A,B). C9ORF80 bound with dT_30_ at the concentration of 1.3 µM, and exhibited 100% binding at the concentration of 85 µM, with a *K*_d_ value of 13.2 ± 1.2 µM; however, there is no binding for C9ORF80 with rU_30_ even at the highest concentration of 85 µM (Figure 4C). Although this concentration of protein may be physiologically irrelevant, it suggests that C9ORF80 binds ssDNA poorer than hNABPs. Because C9ORF80 binds with ssDNA, we determined if C9ORF80 could also bind with random DNA sequence. Hence, we evaluated the binding of the protein with a random 30-mer ssDNA and results revealed that the C9ORF80 exhibited similar binding affinity (Supplementary Figure S5A), ∼19% binding at 12.8 µM. When evaluated with different length of ssDNA under a same molecular ratio of protein : DNA, the minimal length required for successful binding with C9ORF80 is 20 nucleotides (Supplementary Figure S5B) and further increasing the concentration of protein exhibited multiple bands, especially with dT_90_ (Supplementary Figure S5B). However, C9ORF80 did not bind dsRNA (Supplementary Figure S5C), dsDNA (Supplementary Figure S5D), or DNA : RNA hybrid (Supplementary Figure S5E).

### The N-termini of INTS3 are involved in protein–protein interaction, and C-termini in ssDNA/ssRNA binding

**Figure 5.**
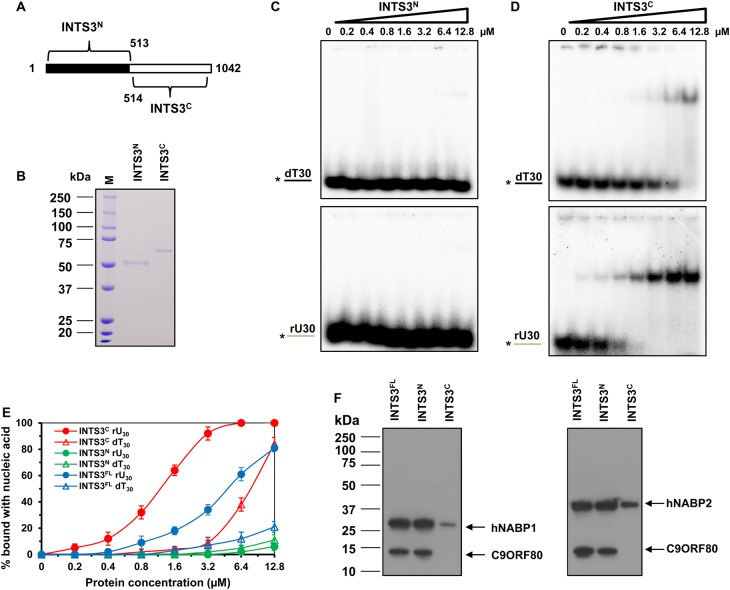
Role of N- and C-terminal regions of INTS3 in nucleic acid-binding and protein–protein interaction. (**A**) Schematic representations of N-terminal and C-terminal region of INTS3. (**B**) SDS–PAGE analysis of the purified N- and C-terminal proteins eluted from size-exclusion chromatography. (**C**) Representative EMSA images of nucleic acid binding of the INTS3^N^ protein with 0.5 nM of dT_30_ (top) and rU_30_ (bottom). (**D**) Representative EMSA images of nucleic acid binding of the INTS3^C^ protein with 0.5 nM of dT_30_ (top) and rU_30_ (bottom). (**E**) Quantitative analysis of INTS3^N^ and INTS3^C^ binding with dT_30_ and rU_30_ in **C** and **D** along with INTS3^FL^ biding with dT_30_ and rU_30_, shown in Figure 2C. (**F**) *E. coli* extracts expressing GST-INTS3^FL^, GST-INTS3^N^, or GST-INTS3^c^ were incubated with purified C9ORF80 and hNABP1 (left) or C9ORF80 and hNABP2 (right). GST-agarose was used to precipitate GST epitope-binding proteins. The resultant protein samples were Western blotted with antibodies against hNABP1 or hNABP2 and C9ORF80, as indicated.

Although the N-terminal 500 amino acids of INTS3 were reported to be sufficient for the interaction with hNABPs [15], the role of C-terminal region is not clear. Also, the N-terminal region of INTS3 does not bind with ssDNA [15]; however, in our present study, we found that the INTS3^FL^ bound ssRNA and ssDNA. Hence, we predicted the DNA-binding residues of INTS3^FL^ using BindN [30] with 90% of expected specificity. The results suggested that the C-terminal region has more positive predictions for DNA binding (Supplementary Figure S6). To evaluate this, we divided the INTS3 into N-terminal region (INTS3^N^, 1–513 amino acids) and C-terminal region (INTS3^C^; 514–1042 amino acids) (Figure 5A). We cloned INTS3^N^ in pGEX-6P-1 and INTS3^C^ in pET28a vectors, and purified the proteins to near homogeneity using affinity and gel filtration (Figure 5B). When evaluated for the nucleic acid binding, INTS3^N^ exhibited poor binding with dT_30_ and rU_30_ (Figure 5C), while INTS3^C^ could bind efficiently with dT_30_ and rU_30_ (Figure 5D), with *K*_d_ value of 7.2 ± 1.2 µM for dT_30_ and 1.2 ± 0.8 µM for rU_30_ (Figure 5E).

Earlier studies have shown that the N-terminal region of INTS3 interacts with hNABP2 and C9OFR80 proteins [15], but the function of the C-terminus is not known. To address this, the GST-tagged INTS3^FL^, INTS3^N^, and INTS3^C^ proteins were purified with glutathione sepharose beads and their interactions with hNABP1/2 and C9ORF80 were evaluated. The results suggested that the INTS3^N^ is primarily involved in the interaction with hNABP1/2 and C9ORF80 (Figure 5F); however, INTS3^C^ also exhibited interaction with hNABP1/2, but not with C9ORF80. Taken together, our results suggest that the N-terminus of INTS3 is majorly involved in protein–protein interaction, and the C-terminus in nucleic acid binding.

### INTS3–hNABP1/2–C9ORF80 heterotrimeric complex has reduced binding affinity with ssDNA and ssRNA

**Figure 6.**
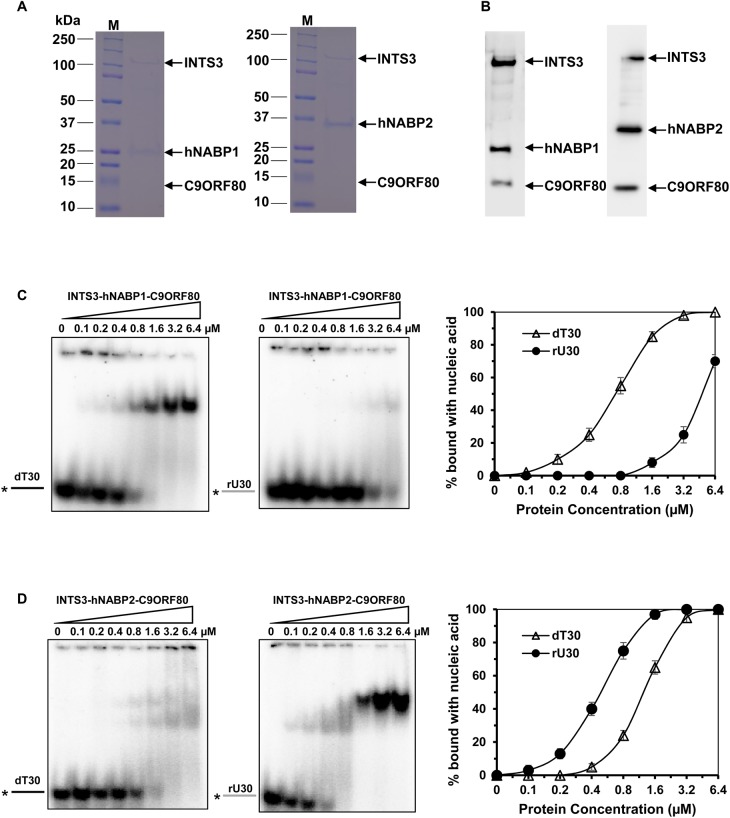
Purification and binding ability of INTS3–hNABP1/2–C9ORF80 heterotrimeric complex. (**A**) SDS–PAGE analysis of co-expressed INTS3–hNABP1–C9ORF80 (left) and INTS3–hNABP2–C9ORF80 (right) complex purified with Glutathione sepharose followed by Ni-NTA chromatography. (**B**) Western blot analysis for the purified INTS3–hNABP1–C9ORF80 (left) and INTS3–hNABP2–C9ORF80 (right) complex using respective antibodies. (**C**) Representative EMSA images of INTS3–hNABP1–C9ORF80 complex binding with dT_30_ (left), rU_30_ (middle), and their quantitative analysis (right). (**D**) Representative EMSA images of INTS3–hNABP2–C9ORF80 complex binding with dT_30_ (left), rU_30_ (middle), and their quantitative analysis (right).

Because INTS3–hNABP1/2–C9ORF80 exists as a heterotrimeric complex *in vivo*, next we wished to investigate the nucleic acid-binding ability of the heterotrimer. Attempt has been made to purify the heterotrimer from insect cells; however, the yield and purity is not ideal [7]. In the present study, we tagged INTS3^FL^ with a GST, and hNABP1/2, as well as C9ORF80, with His, and the vectors were co-transformed in bacteria. The three proteins were co-expressed in bacteria and the complex was purified by two-step affinity chromatography: glutathione sepharose beads followed by Ni-NTA beads. We have successfully purified the INTS3^FL^–hNABP1/2–C9ORF80 complex (Figure 6A), suggesting that these three subunits associate with each other *in vivo*. Although the C9ORF80 protein is not evidently visible on SDS–PAGE gel, we could detect the protein by Western blot (Figure 6B).

To evaluate the binding ability of the INTS3^FL^–hNABP1/2–C9ORF80 protein complex, we incubated the complexes independently with ssDNA (dT_30_) or ssRNA (rU_30_), and found that the INTS3^FL^–hNABP1–C9ORF80 complex exhibited higher binding affinity with dT_30_ than rU_30_ (Figure 6C). For instance, at the concentration of 0.1 µM, the complex could bind dT_30_, while at the concentration of 1.6 µM it bound rU_30_. On the other hand, INTS3^FL^–hNABP2–C9ORF80 complex exhibited higher affinity toward rU_30_ than dT_30_ (Figure 6D), it bound dT_30_ at 0.4 µM while it bound rU_30_ at 0.1 µM. This substrate specificity of the complex is similar to that of hNABP1 and hNABP2 protein alone [18], where hNABP1 has higher affinity toward ssDNA and hNABP2 has higher affinity toward ssRNA. To confirm that the INTS3^FL^–hNABP1/2–C9ORF80 complex, not the dissociated hNABP1/2 proteins, is binding to the substrate, we incubated dT_30_ with an increasing concentration of INTS3^FL^–hNABP1–C9ORF80 complex (Supplementary Figure S7A), and rU_30_ with increasing concentration of INTS3^FL^–hNABP2–C9ORF80 complex (Supplementary Figure S7B). The protein/nucleic acid complex was transferred to the nitrocellulose membrane, and proteins were detected with INTS3, hNABP1/2, and C9ORF80 antibodies. All three proteins, INTS3, hNABP1/2, and C9ORF80, were present in the complex (∼250 kDa), but INTS3 and C9ORF80 were not observed in the hNABP1/2 control, indicating that the complex itself is binding to the substrate. However, the complex exhibits reduced affinity compared with their individual hNABP1/2 proteins. For example, INTS3–hNABP1–C9ORF80 complex has a *K*_d_ value of 0.6 ± 0.09 µM with ssDNA (Figure 6C), while hNABP1 has a *K*_d_ value of 0.15 ± 0.04 µM (Supplementary Figure S8A). Similarly, the INTS3^FL^–hNABP2–C9ORF80 complex has a *K*_d_ value of 0.5 ± 0.07 µM with ssRNA (Figure 6D), while the NABP2 has a *K*_d_ value of 0.06 ± 0.02 µM (Supplementary Figure S8B).

### INTS3, but not C9ORF80, affects the nucleic acid binding of hNABP1 and hNABP2

**Figure 7.**
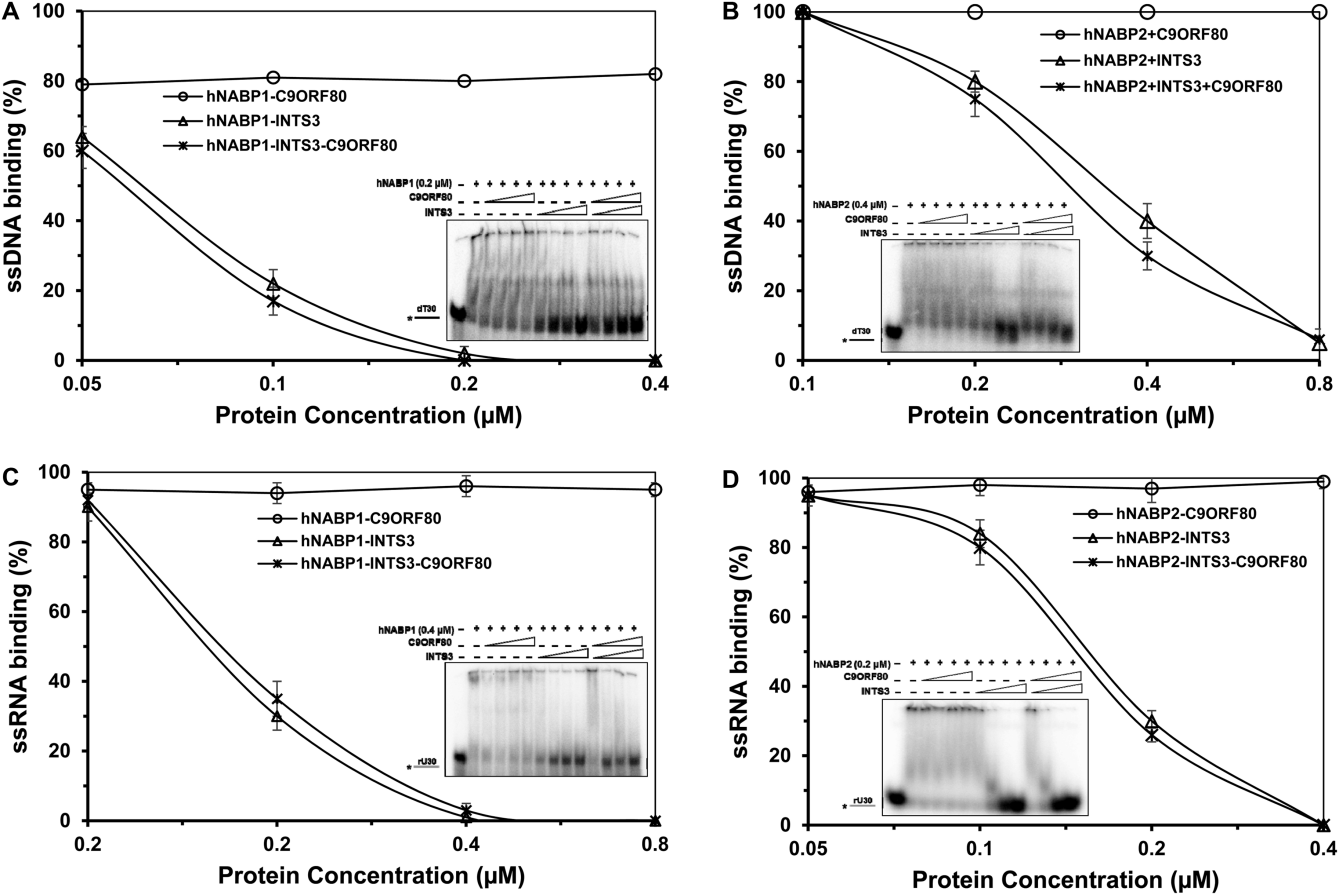
Reconstituted INTS3, hNABP1/2, and C9ORF80 protein complex binding with nucleic acid. Quantitative analysis of reconstituted INTS3, hNABP1, and C9ORF80 protein complex (**A** and **C**), INTS3, hNABP2, and C9ORF80 protein complex (**B** and **D**) binding with dT_30_ (**A** and **B**) or rU_30_ (**C** and **D**). Insets are representative EMSA images.

Since INTS3–hNABP1/2–C9ORF80 complex protein could bind ssDNA and ssRNA but with reduced affinity, we asked which one, INTS3 or C9ORF80, affected the binding ability of hNABP1 and hNABP2. Thus, we reconstituted this heterotrimeric complex by mixing them at various concentrations, and it was subjected to EMSA. hNABP1 (Figure 7A) and hNABP2 (Figure 7B) exhibited 80 and 100% binding, respectively, with the ssDNA (dT_30_) at a concentration of 0.2 and 0.4 µM, respectively. C9ORF80 did not affect hNABPs' binding ability with ssDNA, even at the ratio of 2 : 1 with hNABPs. This suggests that C9ORF80 is not critical for hNABP binding with ssDNA. On the contrary, INTS3 inhibited the binding ability of hNABPs with ssDNA at the ratio of 1 : 1 with hNABP. Further increasing the concentration of INTS3, it completely inhibited the DNA-binding ability of hNABPs.

Similar to the DNA-binding results, C9ORF80 did not affect the binding ability of hNABP proteins with ssRNA (Figure 7C,D). However, INTS3 protein inhibited the binding ability of hNABPs with ssRNA, where at the respective concentration of 0.2 and 0.4 µM, hNABP1 (Figure 7C) and hNABP2 (Figure 7D) exhibited 95% binding with the 30-mer RNA. In conclusion, C9ORF80 has no effect on hNABPs' binding ability with DNA or RNA, but INTS3 plays a vital role for the binding between hNABPs and nucleic acids.

## Discussion

The hNABP1/2 associates independently with INTS3 and C9ORF80 proteins [7,8,16]; however, the biochemical functionality of these two subunits is not known. Here, we demonstrate that INTS3, but not C9ORF80, affects the binding ability of hNABP1 and hNABP2, indicating that INTS3 might regulate hNABP1 and hNABP2's biological function.

The RPA heterotrimer consists of RPA70, RPA32, and RPA14 subunits, with three OB-fold in RPA70 subunit and one in each of the RPA34 and RPA14 subunits. Previously, we have shown that hNABPs have a significant lower affinity to DNA than RPA [18], which might be due to the fact that RPA is composed of five OB-folds, while hNABPs have one. Compared with RPA, a significant higher ratio of protein : DNA is required for hNABPs DNA or RNA binding, whereas similar observation has been reported [6]. However, OB-fold is present only in hNABP subunit in hNABP–INTS3–C9ORF80 heterotrimeric complex. Given that the other two binding partners — INTS3 and C9ORF80 — do not contain OB-fold, it is likely that even the INTS3–NABP–C9ORF80 complex may not have a significantly higher affinity to nucleic acid. In fact, INTS3–NABP2–C9ORF80 complex purified from the insect cell line exhibits similar binding affinity to ssDNA as hNABP2 alone in a previous study [7]. Consistently, the hNABP1/2 heterotrimeric complex exhibited similar nucleic acid substrate affinity as hNABP1 and hNABP2 alone in the present study, where hNABP1 complex binds with ssDNA and hNABP2 complex binds with ssRNA.

The crystal structure of SOSS1 (sensor of ssDNA) complex (hNABP2, INTS3, and C9ORF80) has revealed that NABP2 protein interacts with nucleic acid and INTS3 via the N-terminus OB-fold, while the C9ORF80 interacts with INTS3, but not with NABP2 [15]. In our present study, we have attempted to express and purify full-length INTS3 using C-terminal 6×His-tag in bacteria and C-terminal 3×FLAG tag in mammalian cells, but with no success. Also, the attempt to purify the INTS3^N^ fragment with C-terminal 6×His-tag was not successful, even though the protein was expressed (data not shown). However, we succeeded in purifying INTS3^FL^ and INTS3^N^ using N-terminal GST-tag, suggesting that the N-terminus of INTS3 might affect the conformation of the full-length INTS3 protein, resulting in inaccessibility of C-terminal tag for efficient binding with the beads in affinity chromatography. This might be the reason why, we found that, INTS3^C^ has 5-fold higher affinity than INTS3^FL^ (Figure 5E), because the predicted nucleic acid-binding domain is predominant in the C-terminus of INTS3 and the N-terminus of INTS3 might affect the accessibility of INTS3^FL^ to nucleic acids. Also, from our present study, the N-terminus of the protein does not exhibit any nucleic acid binding; however, it exhibits direct interaction with hNABP and C9ORF80, and inhibits the C-terminus-binding affinity. Even though the binding affinity of hNABP proteins is reduced by the presence of full-length INTS3 protein, it prevented the multimerization of hNABP1 that is observed in our recent study [18], suggesting that INTS3 might co-ordinate the binding efficiency of hNABP1/2. Thus, the C-terminus of INTS3 is essential for the complete functionality of the protein and might regulate the binding of hNABP proteins toward the substrate.

In our current study, the complex reduced the binding affinity of hNABPs by 5-fold toward ssDNA and ssRNA. INTS3 inhibits the hNABPs' binding with ssDNA and ssRNA. Since the OB-fold of hNABP2 is responsible for the interaction with INTS3 [15,16] and binding with ssDNA, INTS3 might block the ability of hNABP2's binding with ssDNA, where in our present study, increasing concentration of INTS3 has rapidly decreased the binding efficiency of hNABP1 and hNABP2 with the nucleic acids. On the other hand, C9ORF80 did not affect the binding affinity of hNABP proteins, which is consistent with previous reports — there is no direct interaction between NABP and C9ORF80 [7,15,16]. Interestingly, INTS3 and C9ORF80 co-localize with hNABP2 protein at the DNA damage site after hydroxyurea treatment [31], while other studies demonstrate that INTS3 does not localize with single-strand break or double-strand break at least immediately after DNA damage [16,32]. Also, Skaar et al. [16] have shown that INTS3 forms discrete foci independent of DNA damage; moreover, it does not co-localize with hNABP2 or γ-H2AX; however, INTS3 relocates to the DNA damage site at extended time, suggesting that INTS3 might regulate the recruitment of hNABP2 to DNA damage sites. Together with our results, INTS3 might be essential for the recruitment of hNABP2 protein at the DNA damage sites, but may not have a major role in DNA binding of the heterotrimer protein complex.

Recently, the Richard laboratory found that hNABP2 is associated with a large number of proteins with roles in mRNA metabolism and various chromatin-remodeling complexes [33]. Similarly, our co-IP experiment also revealed many transcription-related proteins associate with hNABP2, indicating its potential role in transcriptional regulation (Supplementary Figure S9 and Table S5). Previously, we have shown that hNABP2 has higher affinity towards ssRNA [18] and similar results with the complex where INTS3–hNABP2–C9ORF80 complex has higher affinity with ssRNA than ssDNA. Since the complex exhibits reduced binding affinity than hNABP1/2 and hNABP's association with various transcription factors, we speculate that the complex might mediate damage response when RNA polymerase encounters the DNA damage, for example, the complex might recruit hNABP1/2 to the damage site, leading to transcription termination and trigger the repair mechanism to maintain genome stability.

## Conclusions

We have purified INTS3 and C9ORF80 proteins and found both exist as a monomer in solution. INTS3 binds both ssDNA and ssRNA, but C9ORF80 binds ssDNA only. Moreover, we purified the INTS3–hNABP1/2–C9ORF80 heterotrimeric complex, and found that it binds ssDNA and ssRNA at reduced affinity, compared with hNABP1/2 alone. Collectively, our results indicate that INTS3 might function as a scaffold protein to recruit hNABP1/2 to DNA damage sites to maintain genome integrity.

## Abbreviations

CD: circular dichroism
CPSF100: Cleavage and polyadenylation specificity factor 100 kDa subunit
CV: column volume
dsDNA: double-stranded DNA
dsRNA: double-stranded RNA
EMSA: electrophoretic mobility shift assays
hNABP: human nucleic acid-binding protein
HR: homologous recombination
IP: immunoprecipitation
INTS3: integrator complex subunit 3
NABP1: nucleic acid-binding protein 1
NABP2: nucleic acid-binding protein 2
OB-fold: oligonucleotide/oligosaccharide binding fold
PMSF: phenylmethylsulfonyl fluoride
PONDR: predictor of natural disordered regions
PSIPRED: PSI-blast-based secondary structure PREDiction
RNAPII: RNA polymerase II
RPA: replication protein A
SEC-MALS: size-exclusion chromatography with multi-angle light-scattering
snRNAs: small nuclear RNAs
SSB: single-stranded DNA-binding protein
ssDNA: single-stranded DNA
ssRNA: single-stranded RNA
