# Supplementary material for: Biochemical characterization of INTS3 and C9ORF80, two subunits of hNABP1/2 heterotrimeric complex in nucleic acid binding
Source: Biochem J. 2018 Jan 2;475(1):45–60. doi: 10.1042/BCJ20170351 (PMC5748837; doi:10.1042/BCJ20170351)
Supplement: Supplementary Tables and Figures [file BCJ-475-45-s1.pdf]

Table S1: PCR primers and substrates used in this study

Name	Sequence (5′-3′)	Used in this study
C9ORF80-F-NcoI	GCATCCATGGCAGCAAACTCTTCAGG	Forward primer to PCR amplify C9ORF80 gene
C9ORF80-R-XhoI	CGATCTCGAGTTCTGGGTCAAGGCGAGGTAAAAC	Reverse primer to PCR amplify C9ORF80 gene
INTS3-6P1-F-BamHI	GCATGGATCCATGGAGATGGACAACCATATG	Forward primer to PCR amplify INTS3 gene to clone in pGEX-6P-1 vector
INTS3-6P1-R-XhoI	GCATCTCGAGATCAGTCACTGTCAGAGCCCACTG	Reverse primer to PCR amplify INTS3 gene to clone in pGEX-6P-1 vector
INTS3-N-R-XhoI	GCATCTCGAGGGAAACTGGCTCCTCAATTT	Reverse primer to PCR amplify the N-terminus of INTS3 gene
INTS3-C-F-NdeI	GCATGCTAGCATGGAGATGGACAACCATAT	Forward primer to PCR amplify C-terminus of INTS3 gene to clone in pET28a vector
INTS3-C-R-XhoI	GCATCTCGAGGTCACTGTCAGAGCCC	Reverse primer to PCR amplify C-terminus of INTS3 gene to clone in pET28a vector
INTS3-C-6P1-F-BamH1	TGCAGGATCCATGGAGATGGACAACCATAT	Forward primer to PCR amplify C-terminus of INTS3 gene to clone in pGEX-6P-1 vector
dT$_{10,\ 20,\ 30,\ 60\ or\ 90}$	(dT)$_{n=10,\ 20,\ 30,\ 60\ or\ 90}$	ssDNA
rU$_{30}$	UUUUUUUUUUUUUUUUUUUUUUUUUUUUUU	ssRNA
RNA 30-mer	GAGCUACCAGCUACCCCGUAUGUCAGAGAG	Random sequence ssRNA; form dsRNA with RNA 30-mer comp
DNA 30-mer	GAGCTACCAGCTACCCCGTATGTCAGAGAG	Random sequence ssDNA; form DNA:RNA hybrid with RNA 30-mer comp
DNA 60-mer	ACGCAGTTGAGTATTCTACAGAGTTGGCGT GAGCTACCAGCTACCCCGTATGTCAGAGAG	Random sequence ssDNA
RNA 30-mer comp	CUCUCUGACAUACGGGGUAGCUGGUAGCUC	Complementary strand to RNA 30-mer; form dsRNA with RNA 30-mer; form RNA:DNA hybrid with DNA 30-mer

Restriction digestion sites are underlined.

Table S2: Peptide identification by mass spectrometric in-gel digestion analysis of INTS3 band (Corresponding to 110 kDa on SDS-PAGE, amino acid coverage: 65.3%)

#	Sequence	Protein Group Accessions	Modifications	Parent Charge	Parent Mass to Charge Ratio (m/z) (Da)
1	AAASGAAGGGGGGAGAGAPGGGR	Q68E01		2	806.3853
2	AALSSPR	Q68E01		2	351.2026
3	ACQEDDVR	Q68E01		2	496.7103
4	CDLIR	Q68E01		2	338.6771
5	CmSIVTSmTAGVSER	Q68E01	m:Oxidized methionine	3	554.2525
6	DELEER	Q68E01		2	395.6834
7	DGmNIVLNK	Q68E01	m:Oxidized methionine	2	510.2635
8	DIIHNPQ	Q68E01		2	418.7181
9	DLALVSR	Q68E01		2	387.2303
10	EAnDALNAYVCK	Q68E01	n:Deamidated	2	684.8197
11	EANDALNAYVCK	Q68E01		3	456.5484
12	EKPSEEmVK	Q68E01	m:Oxidized methionine	3	364.8488
13	EWVLK	Q68E01		2	337.6981
14	FHPIK	Q68E01		2	321.1942
15	FLACR	Q68E01		2	333.6742
16	GAAAAAAASGAAGGGGGGAGAGAPGGGR	Q68E01		2	976.9698
17	HDELLAEHIK	Q68E01		3	402.2185
18	IEEPVSmEmDNHmSDK	Q68E01	m:Oxidized methionine	3	647.2655
19	IEEPVSmEmDnHmSDK	Q68E01	m:Oxidized methionine n:Deamidated	3	647.5988
20	ILTEPAQAQK	Q68E01		2	549.8131
21	INQILmEK	Q68E01	m:Oxidized methionine	2	502.7764
22	INQILMEk	Q68E01	k:Carbamylated lysine	2	516.2829
23	INQILMEK	Q68E01		2	494.7798
24	InQILmEK	Q68E01	n:Deamidated m:Oxidized methionine	2	503.2734
25	InQILMEK	Q68E01	n:Deamidated	2	495.2799

26	INQILMEk	Q68E01	k:Carbamylated lysine	2	516.2829
27	LAPLFDNPK	Q68E01		2	507.7853
28	LDESLR	Q68E01		2	366.7
29	LLFmTSR	Q68E01	m:Oxidized methionine	2	442.2395
30	LLLSTSLDAK	Q68E01		2	530.8173
31	LLQNVAR	Q68E01		2	407.2517
32	LLQSR	Q68E01		2	308.6944
33	LLTTCTSNVAASNAK	Q68E01		2	775.8981
34	LNLTNTK	Q68E01		2	402.2367
35	LQDTCR	Q68E01		2	396.688
36	LTPDMETK	Q68E01		2	467.7325
37	LTPDmETK	Q68E01	m:Oxidized methionine	2	475.73
38	LTPDMETk	Q68E01	k:Carbamylated lysine	2	489.2362
39	NATQPPNAEEESGSSSASEEEDTKPKPTK	Q68E01		4	762.1014
40	NNSLPR	Q68E01		2	350.6926
41	QYLSTPDSQSLR	Q68E01		3	465.5719
42	qYLSTPDSQSLR	Q68E01	q:Pyroglutamic acid	2	689.3359
43	SGVLGADGVCmTFmK	Q68E01	m:Oxidized methionine	2	802.8621
44	SSDILPR	Q68E01		2	394.2207
45	SSLNHIVEK	Q68E01		3	342.8594
46	SSPSPPVEVK	Q68E01		2	513.7774
47	TAGVSER	Q68E01		2	360.1897
48	TLILTEPAQAQK	Q68E01		2	656.8798
49	TQLVWLVR	Q68E01		2	507.8107
50	TSNVAASNAK	Q68E01		2	481.7503
51	VAASNAK	Q68E01		2	330.69
52	VLAHLAPLFDNPK	Q68E01		3	478.9437
53	VLQLQK	Q68E01		2	364.739
54	YQDWFQR	Q68E01		2	521.7396

Table S3: Peptide identification by mass spectrometric in-gel digestion analysis of INTS3 band (Corresponding to 60 kDa on SDS-PAGE, amino acid coverage: 35.6%)

#	Sequence	Protein Group Accessions	Modifications	Parent Charge	Parent Mass to Charge Ratio (m/z) (Da)
1	AAASGAAGGGGGGAGAGAPGGGR	Q68E01		2	806.3853
2	AALSSPR	Q68E01		2	351.2026
3	ACQEDDVR	Q68E01		2	496.7103
4	CDLIR	Q68E01		2	338.6771
5	CmSIVTSmTAGVSER	Q68E01	m:Oxidized methionine	3	554.2525
6	DELEER	Q68E01		2	395.6834
7	DGmNIVLNK	Q68E01	m:Oxidized methionine	2	510.2635
8	DIIHNPQ	Q68E01		2	418.7181
9	DLALVSR	Q68E01		2	387.2303
10	EAnDALNAYVCK	Q68E01	n:Deamidated	2	684.8197
11	EANDALNAYVCK	Q68E01		3	456.5484
12	EKPSEEmVK	Q68E01	m:Oxidized methionine	3	364.8488
13	EWVLK	Q68E01		2	337.6981
14	FHPIK	Q68E01		2	321.1942
15	FLACR	Q68E01		2	333.6742
16	GAAAAAAASGAAGGGGGGAGAGAPGGGR	Q68E01		2	976.9698
17	HDELLAEHIK	Q68E01		3	402.2185
18	IEEPVSmEmDNHmSDK	Q68E01	m:Oxidized methionine	3	647.2655
19	IEEPVSmEmDnHmSDK	Q68E01	m:Oxidized methionine n:Deamidated	3	647.5988
20	ILTEPAQAQK	Q68E01		2	549.8131
21	INQILmEK	Q68E01	m:Oxidized methionine	2	502.7764
22	INQILMEk	Q68E01	k:Carbamylated lysine	2	516.2829
23	INQILMEK	Q68E01		2	494.7798
24	InQILmEK	Q68E01	n:Deamidated m:Oxidized methionine	2	503.2734
25	InQILMEK	Q68E01	n:Deamidated	2	495.2799

26	INQILMEk	Q68E01	k:Carbamylated lysine	2	516.2829
27	LAPLFDNPK	Q68E01		2	507.7853
28	LDESLR	Q68E01		2	366.7
29	LLFmTSR	Q68E01	m:Oxidized methionine	2	442.2395
30	LLLSTSLDAK	Q68E01		2	530.8173
31	LLQNVAR	Q68E01		2	407.2517
32	LLQSR	Q68E01		2	308.6944
33	LLTTCTSNVAASNAK	Q68E01		2	775.8981
34	LNLTNTK	Q68E01		2	402.2367
35	LQDTCR	Q68E01		2	396.688
36	LTPDMETK	Q68E01		2	467.7325
37	LTPDmETK	Q68E01	m:Oxidized methionine	2	475.73
38	LTPDMETk	Q68E01	k:Carbamylated lysine	2	489.2362
39	NATQPPNAEEESGSSSASEEEDTKPKPTK	Q68E01		4	762.1014
40	NNSLPR	Q68E01		2	350.6926
41	QYLSTPDSQSLR	Q68E01		3	465.5719
42	qYLSTPDSQSLR	Q68E01	q:Pyroglutamic acid	2	689.3359
43	SGVLGADGVCmTFmK	Q68E01	m:Oxidized methionine	2	802.8621
44	SSDILPR	Q68E01		2	394.2207
45	SSLNHIVEK	Q68E01		3	342.8594
46	SSPSPPVEVK	Q68E01		2	513.7774
47	TAGVSER	Q68E01		2	360.1897
48	TLILTEPAQAQK	Q68E01		2	656.8798
49	TQLVWLVR	Q68E01		2	507.8107
50	TSNVAASNAK	Q68E01		2	481.7503
51	VAASNAK	Q68E01		2	330.69
52	VLAHLAPLFDNPK	Q68E01		3	478.9437
53	VLQLQK	Q68E01		2	364.739
54	YQDWFQR	Q68E01		2	521.7396

Table S4: Peptide identification by mass spectrometric in-gel digestion analysis of C9ORF80 (amino acid coverage: 80.7%)

#	Sequence	Protein Group Accessions	Parent Charge	Parent Mass to Charge Ratio (m/z) (Da)
1	VAILAELDK	Q9NRY2	2	486.294
2	AILAELDKEK	Q9NRY2	2	565.328
3	LILPVLPR	Q9NRY2	2	460.8208
4	VAILAELDKEK	Q9NRY2	3	410.2454
5	GNLILPVLPR	Q9NRY2	2	546.353
6	ILPVLPR	Q9NRY2	2	404.2774
7	AALQHA	Q9NRY2	2	305.67
8	AALQHAHAH	Q9NRY2	3	319.1697
9	SIALSR	Q9NRY2	2	323.6992
10	AANSSGQGFQNK	Q9NRY2	3	403.528
11	NSSGQGFQNK	Q9NRY2	2	533.7514
12	AANSSGQGFQNKNR	Q9NRY2	3	493.5774
13	DHAEQQH	Q9NRY2	2	432.6861
14	ILPVLPR	Q9NRY2	2	404.2774
15	HPGASIALSR	Q9NRY2	2	504.783
16	SSTNHPGASIALSR	Q9NRY2	2	699.362
17	HIAAQQK	Q9NRY2	2	398.229
18	PGASIALSR	Q9NRY2	2	436.2549
19	QHIAAQQK	Q9NRY2	2	462.2581
20	ANSSGQGFQNK	Q9NRY2	2	569.2695
21	ILPVLPR	Q9NRY2	2	404.2774
22	AALQHAH	Q9NRY2	2	374.1996
23	ASIALSR	Q9NRY2	2	359.2183
24	LLMQNQSSTNHPGAS	Q9NRY2	3	528.9217
25	STNHPGASIALSR	Q9NRY2	3	437.5677
26	DHAEQQHIAAQ	Q9NRY2	2	624.2934
27	QSSTNHPGASIALSR	Q9NRY2	2	763.3917
28	AALQHAHAHSSGY	Q9NRY2	3	450.552
29	DFRDHAEQQHIAAQQK	Q9NRY2	4	481.2411
30	DHAEQQHIAAQQK	Q9NRY2	2	752.3689
31	TNHPGASIALSR	Q9NRY2	3	408.5565

Table S5: Mass spectrometric analysis of proteins pull down with an anti-hNABP2 antibody

Accession	Description	Score	Coverage	# Peptides	MW [kDa]
P25398	40S ribosomal protein S12	4.13	15.15	2	14.5
P62277	40S ribosomal protein S13	13.78	16.56	3	17.2
P62263	40S ribosomal protein S14	9.99	23.84	4	16.3
P62244	40S ribosomal protein S15a	6.31	13.85	2	14.8
P62249	40S ribosomal protein S16	22.12	47.26	7	16.4
P62269	40S ribosomal protein S18	45.63	48.68	9	17.7
P39019	40S ribosomal protein S19	11.3	17.93	3	16.1
P60866	40S ribosomal protein S20	5.49	19.33	2	13.4
P62266	40S ribosomal protein S23	5.36	13.29	2	15.8
P62847	40S ribosomal protein S24	5.82	20.3	2	15.4
P62851	40S ribosomal protein S25	4.61	8.8	2	13.7
P62854	40S ribosomal protein S26	6.7	20.87	2	13
P05388	60S acidic ribosomal protein P0	11.76	17.67	4	34.3
P05387	60S acidic ribosomal protein P2	9.17	53.04	3	11.7
P30050	60S ribosomal protein L12	10.68	24.24	3	17.8
P35268	60S ribosomal protein L22	10.8	38.28	4	14.8
P62829	60S ribosomal protein L23	10.08	32.14	3	14.9
P61353	60S ribosomal protein L27	4.29	12.5	2	15.8
P62888	60S ribosomal protein L30	13.71	34.78	4	12.8
P62899	60S ribosomal protein L31	7.07	18.4	2	14.5
P62910	60S ribosomal protein L32	7.88	20	2	15.8
Q9Y3U8	60S ribosomal protein L36	4.43	20	2	12.2
P61204	ADP-ribosylation factor 3	9.56	19.89	3	20.6
P07355	Annexin A2	5.48	8.26	2	38.6
O95831	Apoptosis-inducing factor 1, mitochondrial	5.24	3.92	2	66.9
P46063	ATP-dependent DNA helicase Q1 (RECQ1)	4.99	3.85	2	73.4
Q9H3F6	BTB/POZ domain-containing adapter for CUL3-mediated RhoA degradation protein 3 (BACD3)	4.87	9.27	2	35.4
Q9UJS0	Calcium-binding mitochondrial carrier protein Aralar2	7.62	6.07	3	74.1
Q16630	Cleavage and polyadenylation specificity factor subunit 6	10.2	7.44	3	59.2
Q8N684	Cleavage and polyadenylation specificity factor subunit 7	6.79	8.07	3	52
Q14258	E3 ubiquitin/ISG15 ligase TRIM25	5.53	3.97	2	70.9
Q15717	ELAV-like protein 1	8.44	10.43	3	36.1
P05198	Eukaryotic translation initiation factor 2 subunit 1	6.32	11.11	3	36.1
P51114	Fragile X mental retardation syndrome-related protein 1	6.14	4.19	2	69.7
P08107	Heat shock 70 kDa protein 1A/1B	26.75	15.91	8	70

P11142	Heat shock cognate 71 kDa protein	67.49	32.2	19	70.9
P08238	Heat shock protein HSP 90-beta	4	3.18	2	83.2
P09651	Heterogeneous nuclear ribonucleoprotein A1	14.97	6.99	2	38.7
P61978	Heterogeneous nuclear ribonucleoprotein K	9.17	8.21	3	50.9
P52272	Heterogeneous nuclear ribonucleoprotein M	33.5	21.64	13	77.5
O43390	Heterogeneous nuclear ribonucleoprotein R	8.56	6	3	70.9
Q9BUJ2	Heterogeneous nuclear ribonucleoprotein U-like protein 1	4.53	4.56	2	95.7
P22626	Heterogeneous nuclear ribonucleoproteins A2/B1	13.32	17	4	37.4
P16403	Histone H1.2	4.58	11.27	2	21.4
Q96KK5	Histone H2A type 1-H	10.05	21.09	3	13.9
O60814	Histone H2B type 1-K	12.45	13.49	3	13.9
P62805	Histone H4	4.44	17.48	2	11.4
Q9NZI8	Insulin-like growth factor 2 mRNA-binding protein 1	40.47	23.92	10	63.4
O00425	Insulin-like growth factor 2 mRNA-binding protein 3	39.62	26.08	11	63.7
P19525	Interferon-induced, double-stranded RNA-activated protein kinase	5.24	4.54	2	62.1
Q07666	KH domain-containing, RNA-binding, signal transduction-associated protein 1	6	5.42	2	48.2
Q4G0J3	La-related protein 7	4.9	4.3	2	66.9
Q8N1G4	Leucine-rich repeat-containing protein 47	4.81	5.15	2	63.4
Q15233	Non-POU domain-containing octamer-binding protein	64.65	22.29	10	54.2
Q7Z3B4	Nucleoporin p54	5.02	4.34	2	55.4
Q6P1J9	Parafibromin	4.09	3.77	2	60.5
P11940	Polyadenylate-binding protein 1	91.72	41.04	22	70.6
Q13310	Polyadenylate-binding protein 4	38.14	15.37	9	70.7
Q6UN15	Pre-mRNA 3'-end-processing factor FIP1	4.28	5.39	2	66.5
Q92841	Probable ATP-dependent RNA helicase DDX17	25.9	16.19	10	80.2
Q99623	Prohibitin-2	4.87	7.02	2	33.3
Q8NCA5	Protein FAM98A	11.42	5.78	3	55.4
Q9P258	Protein RCC2	6.72	6.51	3	56
Q92734	Protein TFG	14.19	17	6	43.4
Q13283	Ras GTPase-activating protein-binding protein 1	38.31	21.46	7	52.1
Q9UN86	Ras GTPase-activating protein-binding protein 2	22.07	13.49	5	54.1
P27694	Replication protein A 70 kDa DNA-binding subunit	7.29	5.52	3	68.1
Q96PK6	RNA-binding protein 14 (RBM14)	7.23	5.83	3	69.4
A0AV96	RNA-binding protein 47 (RBM47)	4.67	4.05	2	64.1
P35637	RNA-binding protein FUS	19.62	11.22	6	53.4
Q13501	Sequestosome-1	23.37	24.09	6	47.7
Q01130	Serine/arginine-rich splicing factor 2	6.31	11.31	2	25.5

P30153	Serine/threonine-protein phosphatase 2A 65 kDa regulatory subunit A alpha isoform	7.55	5.26	3	65.3
Q96HS1	Serine/threonine-protein phosphatase PGAM5, mitochondrial	4.74	6.57	2	32
P37108	Signal recognition particle 14 kDa protein	8.18	28.68	2	14.6
Q9UHB9	Signal recognition particle subunit SRP68	4.97	3.51	2	70.7
P62316	Small nuclear ribonucleoprotein Sm D2	6.2	24.58	3	13.5
**Q9BQ15**	**SOSS complex subunit B1**	**6.82**	**14.69**	**2**	**22.3**
Q15637	Splicing factor 1	4.98	4.07	2	68.3
P51571	Translocon-associated protein subunit delta	5.03	13.87	2	19
P12956	X-ray repair cross-complementing protein 6	41.22	30.05	15	69.8
Q9Y5A9	YTH domain-containing family protein 2	5.63	4.66	2	62.3

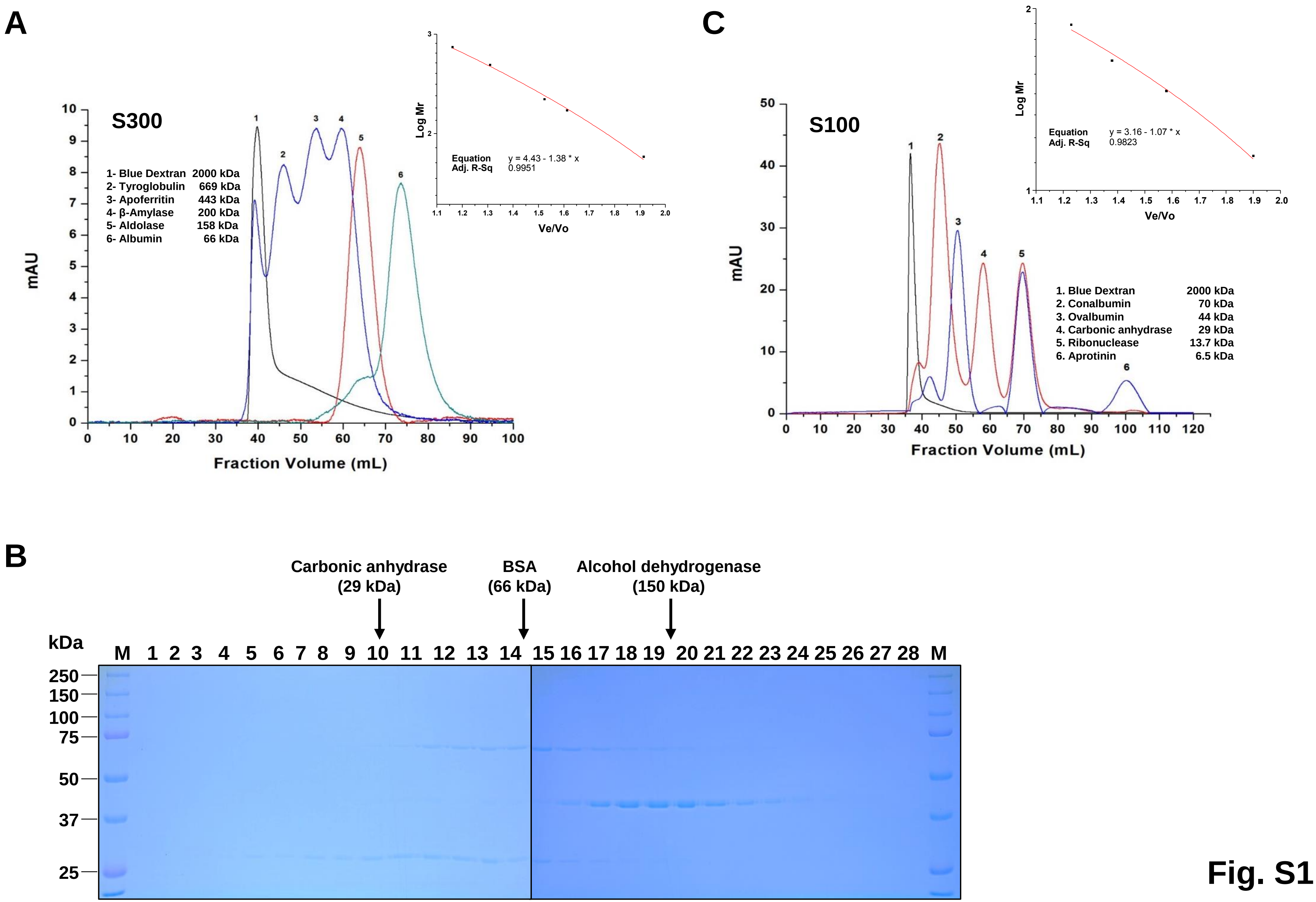
A
S300
1- Blue Dextran 2000 kDa
2- Tyroglobulin 669 kDa
3- Apoferritin 443 kDa
4- β-Amylase 200 kDa
5- Aldolase 158 kDa
6- Albumin 66 kDa
mAU
Fraction Volume (mL)
Log Mr
Ve/Vo
Equation y = 4.43 - 1.38 * x
Adj. R-Sq 0.9951
B
Carbonic anhydrase (29 kDa)
BSA (66 kDa)
Alcohol dehydrogenase (150 kDa)
kDa
M 1 2 3 4 5 6 7 8 9 10 11 12 13 14 15 16 17 18 19 20 21 22 23 24 25 26 27 28 M
250
150
100
75
50
37
25
C
S100
1. Blue Dextran 2000 kDa
2. Conalbumin 70 kDa
3. Ovalbumin 44 kDa
4. Carbonic anhydrase 29 kDa
5. Ribonuclease 13.7 kDa
6. Aprotinin 6.5 kDa
mAU
Fraction Volume (mL)
Log Mr
Ve/Vo
Equation y = 3.16 - 1.07 * x
Adj. R-Sq 0.9823

Fig. S1

Figure S1. (A) Calibration of Sephacryl S-300 HR columns with various standard globular proteins and their calibration curve (right top). (**B**) Coomassie blue stained gels of protein standards (fractions 1-28) collected from sucrose gradient centrifugation. The positions of standards are indicated at the top. Note: Alcohol dehydrogenase is a tetramer containing four equal subunits. (**C**) Calibration of Sephacyl S-100 HR columns with various standard globular proteins and their calibration curve (right top).

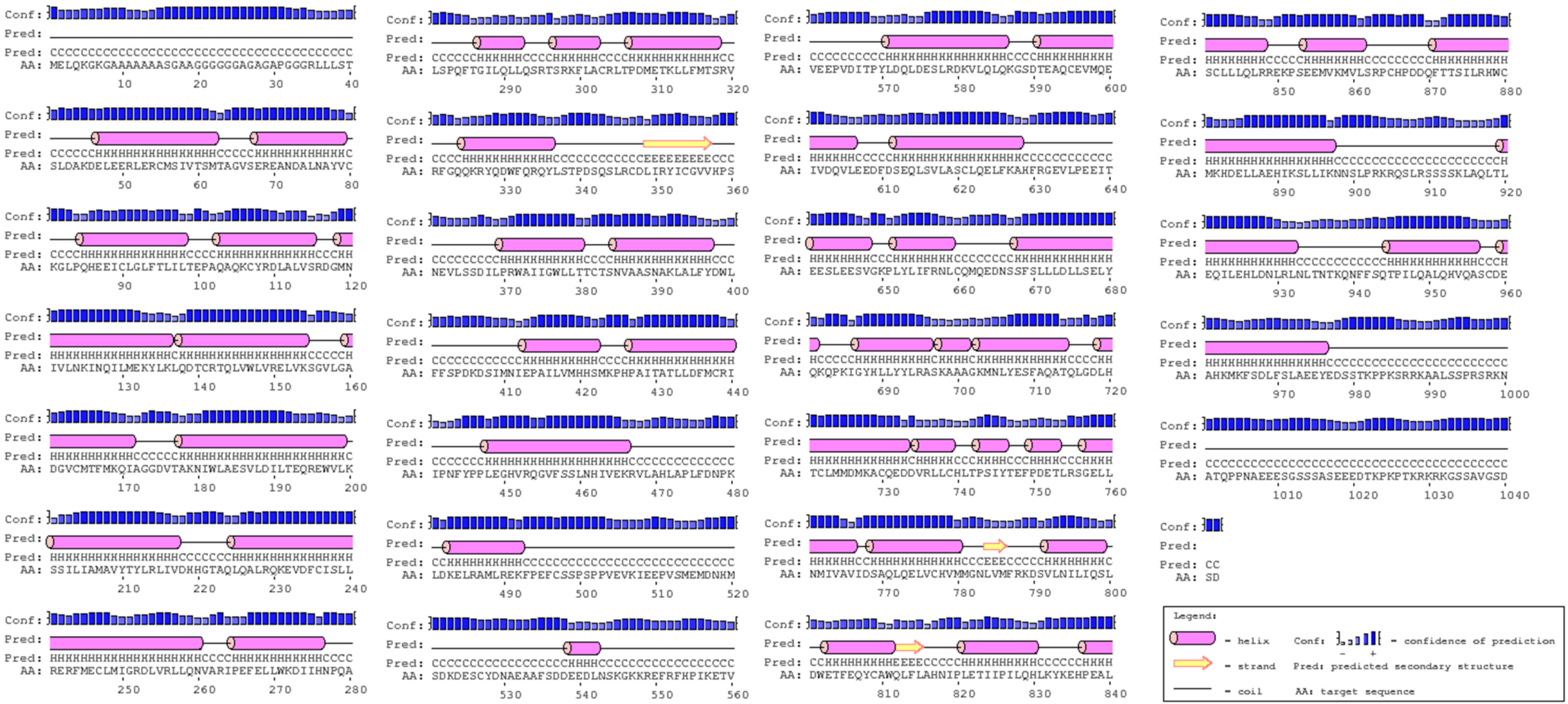

Figure S2. Secondary structure prediction of INTS3 ^{FL} protein by PSIPRED.

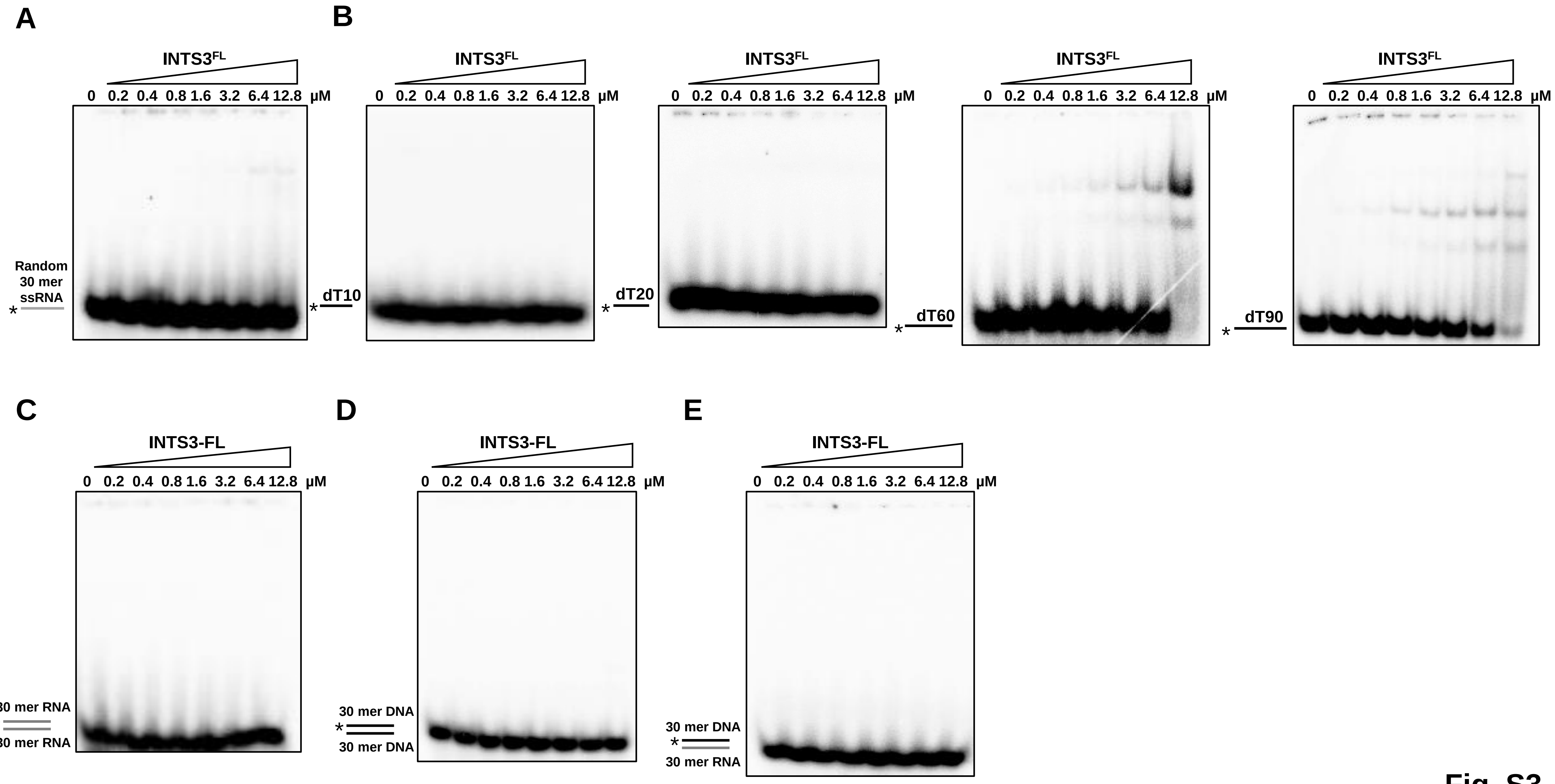
A
B
INTS3^{FL}
0 0.2 0.4 0.8 1.6 3.2 6.4 12.8 μM
Random 30 mer ssRNA
dT10
dT20
dT60
dT90
C
D
E
INTS3-FL
30 mer RNA
30 mer RNA
30 mer DNA
30 mer DNA
30 mer DNA
30 mer RNA

Fig. S3

Figure S3. Representative EMSA images for INTS3 full length protein binding with random 30 mer ssRNA (**A**), various lengths of dT (10, 20, 60 and 90, **B**), dsRNA (**C**), dsDNA (**D**), or DNA: RNA hybrid (**E**).

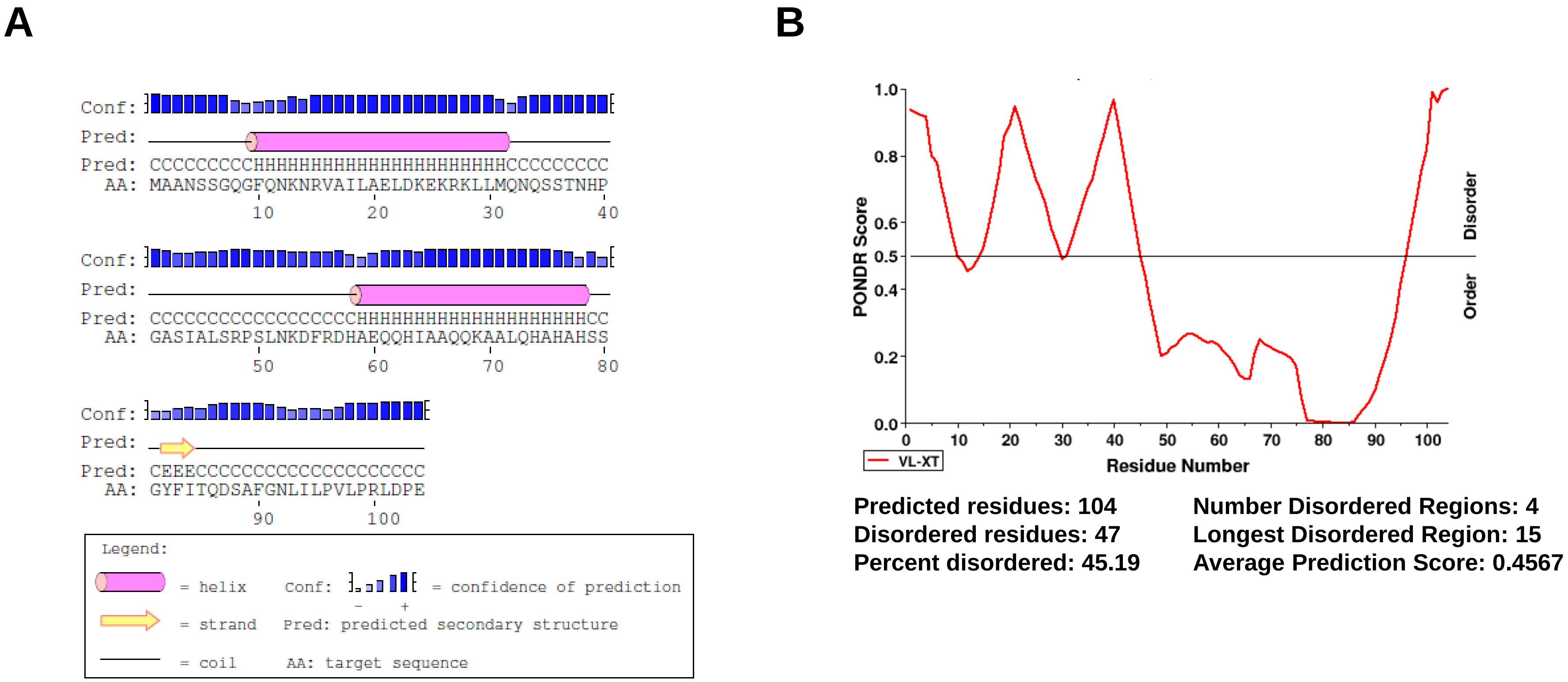

Figure S4. Secondary structure prediction of C9OFR80 protein using PSIPRED (**A**) and PONDR (**B**) showing 45% of random coil structure of the protein.

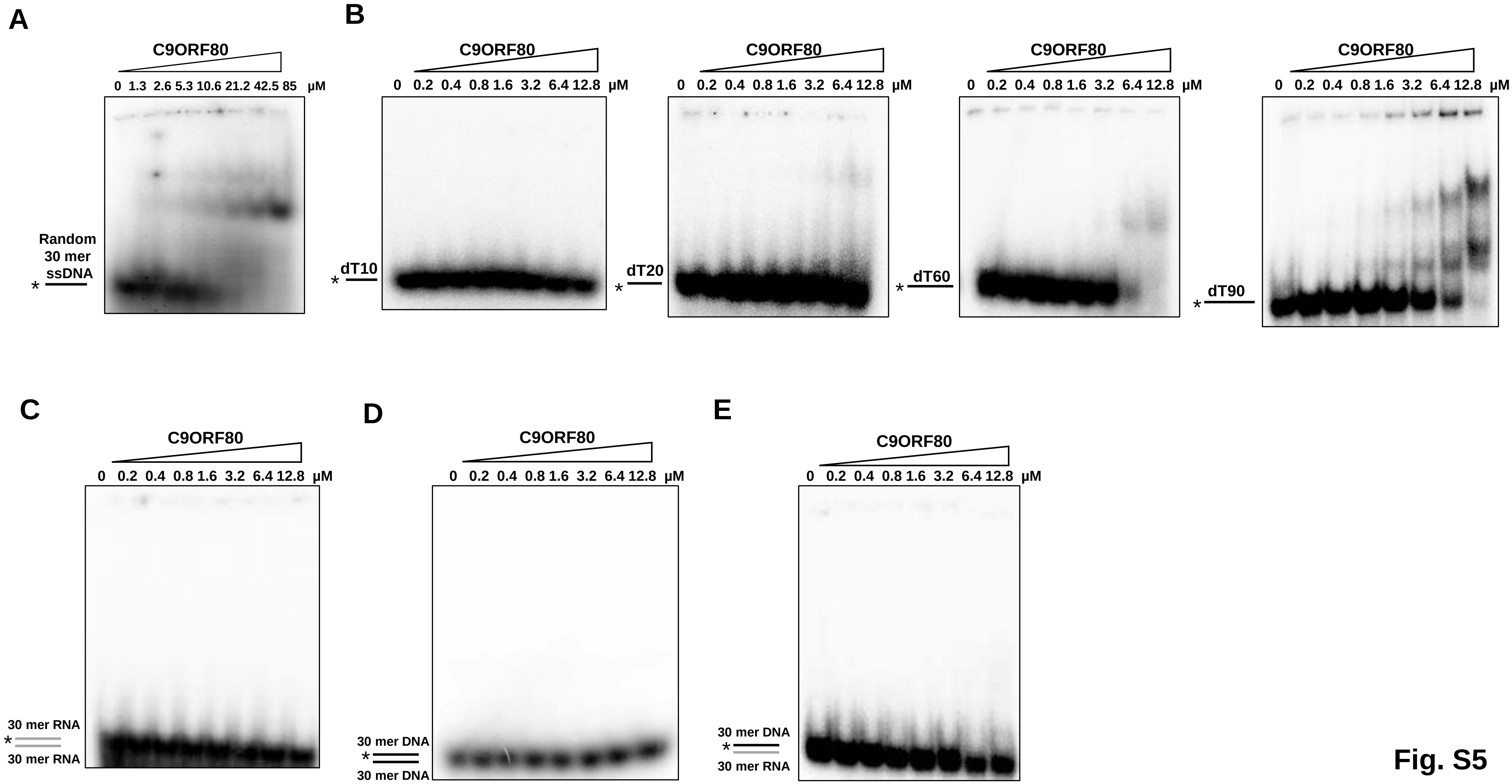
A
C9ORF80
0 1.3 2.6 5.3 10.6 21.2 42.5 85 μM
Random 30 mer ssDNA
*
B
C9ORF80
0 0.2 0.4 0.8 1.6 3.2 6.4 12.8 μM
dT10
dT20
dT60
dT90
C
C9ORF80
0 0.2 0.4 0.8 1.6 3.2 6.4 12.8 μM
30 mer RNA
30 mer RNA
D
C9ORF80
0 0.2 0.4 0.8 1.6 3.2 6.4 12.8 μM
30 mer DNA
30 mer DNA
E
C9ORF80
0 0.2 0.4 0.8 1.6 3.2 6.4 12.8 μM
30 mer DNA
30 mer RNA

Fig. S5

Figure S5. Representative EMSA images for increasing protein concentration of C9ORF80 protein binding with random 30 mer ssDNA (**A**), various lengths of dT (10, 20, 60 and 90, **B**), dsRNA (**C**), dsDNA (**D**), or DNA: RNA hybrid (**E**).

```
Sequence:     MELQKGKGAAAAAAASGAAGGGGGGAGAGAPGGRLLLSTSLDAKDELEERLERCMSIVT
Prediction:   ----+-+--------+-------+------++--+------------------+------
Confidence:   847274823332444722211116111111762394452214551577674966862763

Sequence:     SMTAGVSEREANDALNAYVCKGLPQHEEICLGLFTLILTEPAQAQKCYRDLALVSRDGMN
Prediction:   --------+-----------+---------------------+-++--+------+----
Confidence:   352215126462577373866555477898999939994454647831687567374753

Sequence:     IVLNKINQILMEKYLKLQDTCRTQLVWLVRELVKSGVLGADGVCMTFMKQIAGGDVTAKN
Prediction:   ---------------------+----------------------------------+---
Confidence:   888639438998549262224723964992797125754766877589369845366614

Sequence:     IWLAESVLDILTEQREWVLKSSILIAMAVYTYLRLIVDHHGTAQLQALRQKEVDFCISLL
Prediction:   ---------------------------------------------+--+-----------
Confidence:   948966996963763645815488887993339299986651613636653584899379

Sequence:     RERFMECLMIGRDLVRLLQNVARIPEFELLWKDIIHNPQALSPQFTGILQLLQSRTSRKF
Prediction:   ----------------------+-------------------------------+++++-
Confidence:   283997999991788199348978689699769996762651547279936621987961

Sequence:     LACRLTPDMETKLLFMTSRVRFGQQKRYQDWFQRQYLSTPDSQSLRCDLIRYICGVVHPS
Prediction:   ------------------+-++++-++----------++--+++-+--------------
Confidence:   557183457523874522868668178121155242566218864824791698869453

Sequence:     NEVLSSDILPRWAIIGWLLTTCTSNVAASNAKLALFYDWLFFSPDKDSIMNIEPAILVMH
Prediction:   ------------------------+---+--+----------------------------
Confidence:   446523478638999989833521654361566889899998666353786987999997

Sequence:     HSMKPHPAITATLLDFMCRIIPNFYPPLEGHVRQGVFSSLNHIVEKRVLAHLAPLFDNPK
Prediction:   --------------------------------+---------------------------
Confidence:   535162357273996999399669566844386256523963688539865996886441

Sequence:     LDKELRAMLREKFPEFCSSPSPPVEVKIEEPVSMEMDNHMSDKDESCYDNAEAAFSDDEE
Prediction:   -----+------------------------------------------------------
Confidence:   631587548163864742111225372984783768734624244152648566536775

Sequence:     DLNSKGKKREFRFHPIKETVVEEPVDITPYLDQLDESLRDKVLQLQKGSDTEAQCEVMQE
Prediction:   ---+++++++-+--------------------------+-------+-------------
Confidence:   451697999637564937699898989463995955376537837364131263889979

Sequence:     IVDQVLEEDFDSEQLSVLASCLQELFKAHFRGEVLPEEITEESLEESVGKPLYLIFRNLC
Prediction:   ------------------------------------------------------------
Confidence:   999899998774838288627946972737165975889468486446716918993698

Sequence:     QMQEDNSSFSLLLDLLSELYQKQPKIGYHLLYYLRASKAAAGKMNLYESFAQATQLGDLH
Prediction:   ---------------------+--+---------+--+----+-----------------
Confidence:   363551225289999945823622756159844967173222742636569473374395

Sequence:     TCLMMDMKACQEDDVRLLCHLTPSIYTEFPDETLRSGELLNMIVAVIDSAQLQELVCHVM
Prediction:   ----------------------------------+-------------------------
Confidence:   389899937746759297879343832486554773557859999996363956999798
```

Fig. S6

```
Sequence:     MGNLVMFRKDSVLNILIQSLDWETFEQYCAWQLFLAHNIPLETIIPILQHLKYKEHPEAL
Prediction:   ------------------------------------------------------------
Confidence:   896886734737849994376564886589689999778597499899557122532577

Sequence:     SCLLLQLRREKPSEEMVKMVLSRPCHPDDQFTTSILRHWCMKHDELLAEHIKSLLIKNNS
Prediction:   -------++-+-----------+------------------------------------+
Confidence:   379771667162136792898373533554621279144884997989969226671317

Sequence:     LPRKRQSLRSSSSKLAQLTLEQILEHLDNLRLNLTNTKQNFFSQTPILQALQHVQASCDE
Prediction:   -+++++++++++++----------------+---++------------------------
Confidence:   168898969887773418498699989649771367112126344786399669373653

Sequence:     AHKMKFSDLFSLAEEYEDSSTKPPKSRRKAALSSPRSRKNATQPPNAEEESGSSSASEEE
Prediction:   -------------------+++++++++++--+++++++++++---------+++-----
Confidence:   522827359857986343178999999997119899899968821132321287721112

Sequence:     DTKPKPTKRKRKGSSAVGSDSD
Prediction:   -++++++++++++++---+---
Confidence:   2686999999999898111621 4
```

```
*** Prediction:  binding residues are labeled with '+' and in red;
                 non-binding residues labeled with '-' and in green.
*** Confidence:  from level 0 (lowest) to level 9 (highest).
```

Fig. S6 (cont'd)

Figure S6. Prediction of INTS3 protein binding with ssDNA using BindN with 90% expected specificity.

A

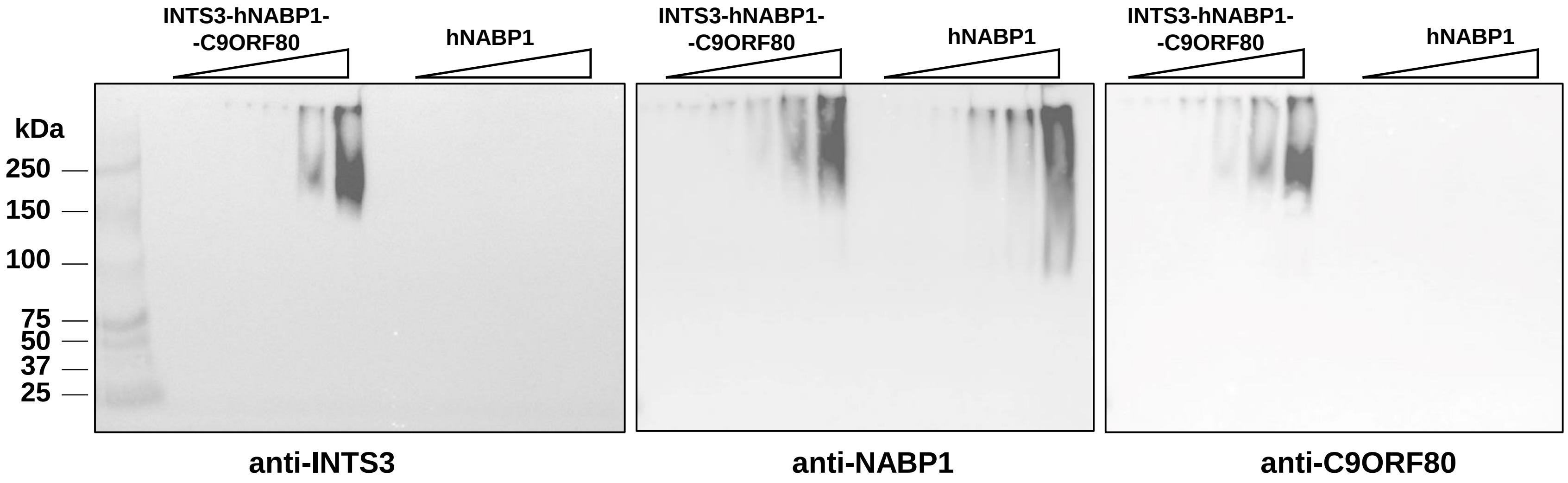
INTS3-hNABP1-
-C9ORF80
hNABP1
INTS3-hNABP1-
-C9ORF80
hNABP1
INTS3-hNABP1-
-C9ORF80
hNABP1
kDa
250
150
100
75
50
37
25
anti-INTS3
anti-NABP1
anti-C9ORF80

B

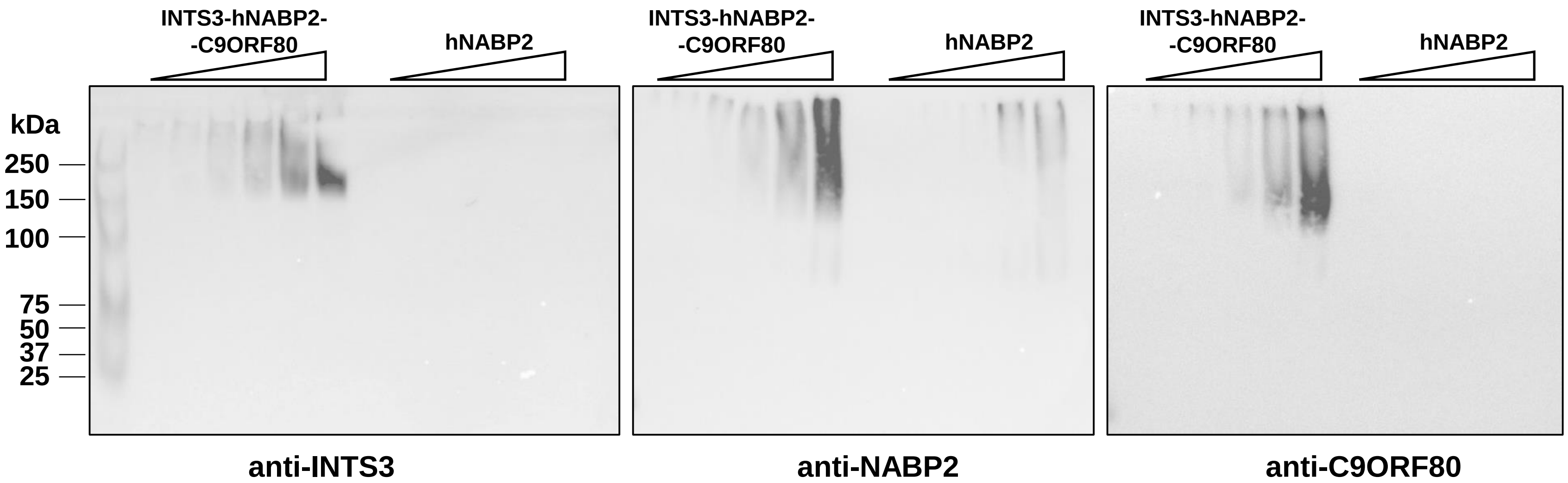
INTS3-hNABP2-
-C9ORF80
hNABP2
INTS3-hNABP2-
-C9ORF80
hNABP2
INTS3-hNABP2-
-C9ORF80
hNABP2
kDa
250
150
100
75
50
37
25
anti-INTS3
anti-NABP2
anti-C9ORF80

Fig. S7

Figure S7. Western blot detection of individual protein with indicated antibodies in the INTS3^{FL}-hNABP1-C9ORF80 complex binding with dT$_{30}$(**A**), and INTS3^{FL}-hNABP2-C9ORF80 complex with rU$_{30}$ (**B**).

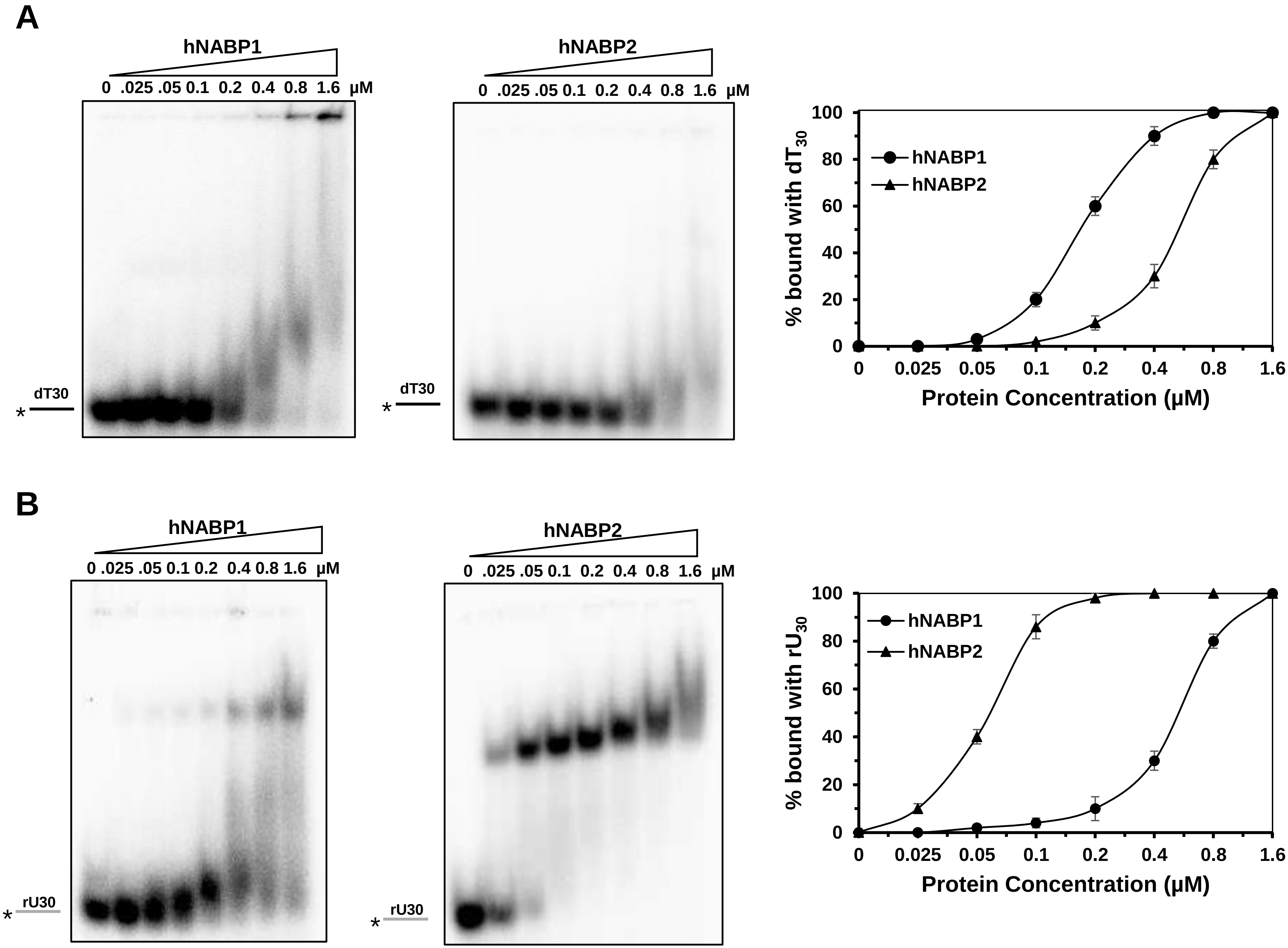
A
hNABP1
0 .025 .05 0.1 0.2 0.4 0.8 1.6 μM
dT30
*
hNABP2
0 .025 .05 0.1 0.2 0.4 0.8 1.6 μM
dT30
*
% bound with dT_{30}
hNABP1
hNABP2
100
80
60
40
20
0
0 0.025 0.05 0.1 0.2 0.4 0.8 1.6
Protein Concentration (μM)
B
hNABP1
0 .025 .05 0.1 0.2 0.4 0.8 1.6 μM
rU30
*
hNABP2
0 .025 .05 0.1 0.2 0.4 0.8 1.6 μM
rU30
*
% bound with rU_{30}
hNABP1
hNABP2
100
80
60
40
20
0
0 0.025 0.05 0.1 0.2 0.4 0.8 1.6
Protein Concentration (μM)

Fig. S8

Figure S8. Representative EMSA images for hNABP1 and hNABP2 binding with dT_{30} (**A**) and rU_{30} (**B**), and their quantitative analysis (right).

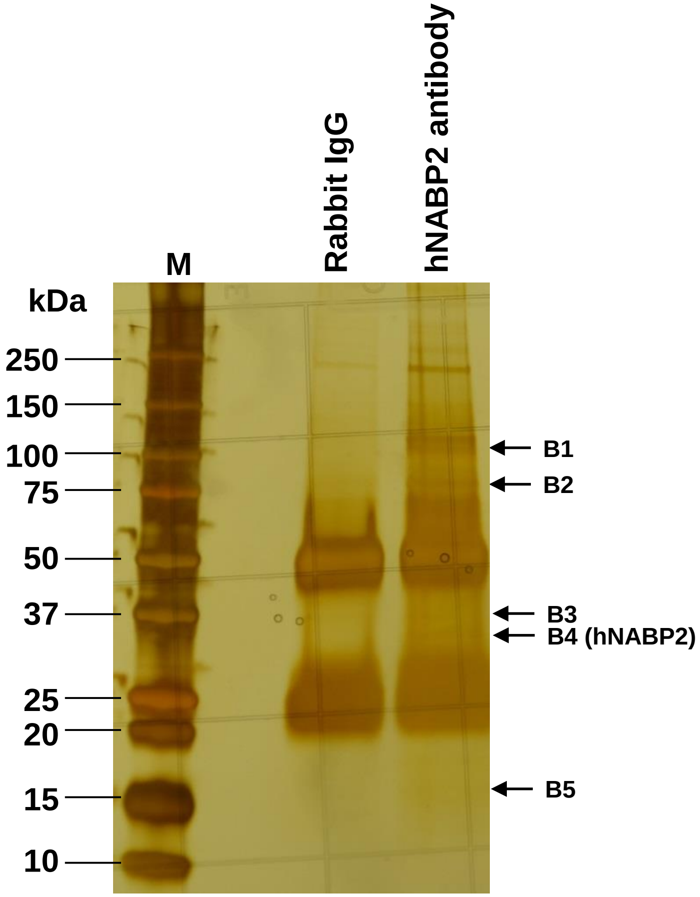

Figure S9. Silver staining of immunoprecipitated proteins using a hNABP2 antibody and rabbit IgG. Samples were separated on a gradient (4-20%) SDS-PAGE gel. M: protein marker. The excised bands are indicated as B1-5.
